# Heparinase Digestion of 3-*O*-Sulfated Sequences: Selective Heparinase II Digestion for Separation and Identification of Binding Sequences Present in ATIII Affinity Fractions of Bovine Intestinal Heparins

**DOI:** 10.3389/fmed.2022.841726

**Published:** 2022-03-31

**Authors:** Pierre Mourier

**Affiliations:** Sanofi Chimie, Aramon, France

**Keywords:** heparinase digestion, heparinase II, 3-*O*-sulfated disaccharides, sulfanilic tagging, bovine intestinal heparin

## Abstract

Binding to antithrombin-III (ATIII) determines the anticoagulant activity of heparin. The complexes formed between heparin and ATIII result from a specific pentasaccharide sequence containing a 3-*O-*sulfated glucosamine in medium position. Building block analysis of heparins, following heparinase digestion, is a critical method in quality control that provides a simple structural characterization of a complex product. Hence, in these applications, study of the digestion of 3-*O*-sulfated moieties merits special attention. With heparinase II, specific inhibition of cleavage of the non-reducing bond of 3-*O*-sulfated units is observed. This specificity was erroneously generalized to other heparinases when it was observed that in exhaustive digests of heparins with the heparinase mixture, resistant 3-*O*-sulfated tetrasaccharides were also obtained from the specific ATIII-binding pentasaccharides. In fact, the detection of unsaturated 3-*O*-sulfated disaccharides in digests of heparin by heparinases I+II+III, resulting from the cleavage of the 3-*O* sulfated unit by heparinase I in non-conventional sequences, shows that this inhibition has exceptions. Thus, in experiments where heparinase II is selectively applied, these sequences can only be digested into tetra- or hexasaccharides where the 3-*O*-sulfated glucosamine is shifted on the reducing end. Heparinase I+II+III and heparinase II digests with additional tagging by reductive amination with sulfanilic acid were used to study the structural neighborhood of 3-*O*-sulfated disaccharides in bovine mucosal heparin fractions with increasing affinity for ATIII. The 3-*O*-sulfated disaccharides detected in heparinase I+II+III digests turn into numerous specific 3-*O*-sulfated tetrasaccharides in heparinase II digests. Additionally, ATIII-binding pentasaccharides with an extra 3-*O*-sulfate at the reducing glucosamine are detected in fractions of highest affinity as heparinase II-resistant hexasaccharides with two consecutive 3-*O*-sulfated units.

## Introduction

Heparin and low molecular weight heparin (LMWH) are linear sulfated polysaccharides which are widely used as anticoagulants for the treatment of thromboembolic disease. The repeating disaccharide units of heparin consist of uronic acid (either iduronic or glucuronic) and glucosamine moieties. The glucosamine can be *N*-sulfated or *N*-acetylated, and positions 2 and 6 of the disaccharide can be *O*-sulfated. 3-*O*-sulfation may also occur infrequently, mostly in association with a pentasaccharidic sequence which specifically binds to antithrombin (ATIII) (1). Taking into account all possible sulfation patterns and acid configurations, 16 different disaccharide units have been found to constitute the building blocks of heparin, with sequencing in the heparin chain determined by a multi-step biosynthesis process involving multiple enzymes in the Golgi apparatus.

Polysaccharide lyases isolated from *Flavobacterium heparinum* cleave glucosamine (1 → 4) uronic acid linkages in heparin without further structural degradation, generating by β-elimination a Δ^4−5^ unsaturated uronic acid with a non-reducing terminal on one fragment and a new reducing end on the other fragment (2, 3). Enzymatic methods have multiple applications in structural investigation of heparins and LMWH. Controlled heparinase depolymerization and oligosaccharide sequencing after isolation by high performance liquid chromatography (HPLC) (4–7) is a useful complement to nuclear magnetic resonance (NMR) for structural determination. When used together, the three heparin lyases (heparinase I [EC 4.2.2.7], heparinase II [no EC number], and heparinase III [EC 4.2.2.8]) can digest all heparinoids into a limited number of building blocks that can be separated by chromatography and quantified (8–11). Eight unsaturated major disaccharide building blocks constitute 80–90% of heparin, with the remaining 10–20% made up of moieties ranging from mono- to tetrasaccharides.

In building block analysis using a mixture of heparinases I, II, and III, the amount of enzyme is sufficient for reaction completion, so the oligosaccharide sequences that result will be resistant to any further hydrolysis. Each heparinase has its own selectivity of cleavage, but when all three are used together, maximum efficiency of cleavage resulting in a simple mixture amenable to straightforward analysis can be achieved. Notably, of the remaining building blocks of so-called exhaustively digested heparin, more than 10 are 3-*O*-sulfated (3S) (3-*O*-sulfation is the main source of heparinase resistance in endogenous heparin). As 3-*O*-sulfation is a key feature of ATIII-binding pentasaccharides, information on the immediate structural environment is particularly valuable. It is clear that the unsaturated 3S tetrasaccharide building blocks (8, 9, 12, 13), which are residues of the ATIII binding pentasaccharides after cleavage on the reducing side (RS) of 3S glucosamine, constitute the major piece of information readily available after digestion of heparins such as from porcine mucosa (PMH), where the binding sites are the well-known “canonical” pentasaccharides [GlcN(NAc/S,6S)-GlcA-GlcN(NS,3S,6S/OH)-IdoA2S-GlcN(NS,6S/OH)].

The digestion of 3S moieties by heparinases has been the subject of several publications (12, 14–17), and the literature indicates that the non-reducing link of the 3S disaccharide cannot be cleaved in ATIII binding pentasaccharides. Thus, the smallest moiety obtainable by digestion with the heparinase mixture is a tetrasaccharide [e.g., ΔIIa-IIs_glu_ (ΔHexUA-GlcNAc(6S)-GlcA-GlcN(NS,3S,6S)] (12, 13) (see Table 1 for structural symbols). Based on available evidence, this resistance to cleavage is generally believed to be due to 3-*O*-sulfation itself, with selectivity demonstrated for heparinase II (18). The literature has suggested that this may also the case for heparinase I, a lyase with a selectivity of cleavage quite different from that of heparinase II (19–22).

**Table 1:** Nomenclature and structural symbols.

**Nomenclature**	
HexUA, Uronic acid	IdoA, L-iduronic acid
GlcA, D-glucuronic acid	ΔHexUA, 4,5-unsaturated uronic acid
GlcN, D-glucosamine	NAc, *N*-acetyl
NS, *N*-sulfate	2S, 2-*O*-sulfate
GalA, D-galacturonic acid	6S, 6-*O*-sulfate
w/w, weight/weight	3S, 3-O-sulfated
**Structural symbols**	
ΔIVa, ΔHexUA-GlcNAc	ΔIVs, ΔHexUA-GlcNS
ΔIIa, ΔHexUA-GlcNAc(6S)	ΔIIIa, ΔHexUA(2S)-GlcNAc
ΔIIs, ΔHexUA-GlcN(NS,6S)	ΔIIIs, ΔHexUA(2S)-GlcNS
ΔIa, ΔHexUA(2S)-GlcNAc(6S)	ΔIs, ΔHexUA(2S)-GlcN(NS,6S)
ΔIIs, ΔHexUA-GlcN(N*S*,3S,6S)	ΔIIIs, ΔHexUA(2S)-GlcN(NS,3S)
ΔIs, ΔHexUA(2S)-GlcN(NS,3S,6S)	IVs_gal_, GalA-GlcNS
IIs_gal_, GalA-GlcN(NS,6S)	IIIs_id_, IdoA(2S)-GlcNS
IIs_glu_, GlcA-GlcN (NS,6S)	Is_id_, IdoA(2S)-GlcN(NS,6S)
IVs_glu_, GlcA-GlcNS	Is_id_, IdoA(2S)-GlcN(NS,3S,6S)
Is_glu_, GlcA(2S)-GlcN(NS,3S,6S)	IIIs_id_, IdoA(2S)-GlcN(NS,3S)
IIs_glu_, GlcA-GlcN(NS,3S,6S)	IVs_glu_, GlcA-GlcN(NS,3S)
Glyserox, Oxidized glycoserine (ΔGlcA-Gal-Gal-Xyl-COOH)	
ΔU(x,y,z), Δ-unsaturated oligosaccharide, x saccharides units, y sulfates, z *N*-acetyl	
U(x,y,z), saturated oligosaccharide, x saccharides units, y sulfates, z *N*-acetyl	
ΔU(x,y,z)^sulf^, ΔU(x,y,z) with sulfanilic acid reductive amination	
G(x,y,z), Oligosaccharide with a glucosamine at its non-reducing end, x saccharides units, y sulfates, z *N*-acetyl	
G(x,y,z)^sulf^, G(x,y,z) with sulfanilic acid reductive amination	
Mw 595^sulf^, Oligosaccharide at Mw595Da with sulfanilic reductive amination (595 + 157Da)	
The iduronic (id) or glucuronic (glu) structure of uronic acids is indicated for oligosaccharides, e.g., ΔIs-III_id_	
Underlined disaccharides have a 3-*O*-sulfated glucosamine, *e.g*. IIs_glu_ (GlcA-GlcNS,3S,6S)	

However, the presence of unsaturated 3S disaccharides ΔIs [ΔHexUA(2S)-GlcN(NS,3S,6S)], ΔIIIs [ΔHexUA(2S)-GlcN(NS,3S)] and ΔIIs [ΔHexUA-GlcN(NS,3S,6S)] among digestion products indicates that the non-reducing side (NRS) of 3S units can be cleaved under some circumstances. These disaccharides have been identified in studies of the action of 3-*O*-sulfotransferases (23–26), which casts doubt on the postulated impossibility of obtaining such structures according to published heparinase specificities (13, 15). We also note that these disaccharides are present in heparin digests when the mixture of heparinases I+II+III is used (4, 8, 9, 27).

In the first part of this study, the cleavage of 3S sequences by heparinases I and II will be updated, and the behavior of heparinase I in particular will be clarified. 3-*O*-sulfation might in fact not be a factor in resistance to heparinase I, i.e., the absence of cleavage at the NRE of the 3S unit in the classical ATIII- binding pentasaccharide structure by heparinase I is due not to 3-*O*-sulfation, but to the presence of the medium 2-OH-glucuronic acid. The description by Chopra et al. (18) of the action of heparinases on 27 synthetic 3S hexasaccharides will be used to characterize the selectivity of heparinase I in this type of moiety. We also aim to demonstrate that 3S unsaturated disaccharides originate not from the “canonical” ATIII binding sites but from ‘non-conventional' sequences based on either 2-*O*-sulfated uronic acid (ΔIs, ΔIIIs) or consecutive 3S disaccharides (ΔIIs), such that their NRS can be cleaved by heparinase I. Unsaturated 3S disaccharides can constitute up to 50% of the total 3S sequences in some bovine mucosal heparins (BMH) (8, 27), and although little information on their structural environment and biological function is available, data suggest that they do not participate in anticoagulation and may be classified as non–ATIII-binding units (17). Recent studies (28, 29) based on biosynthetic oligosaccharides have shown, however, that the glucuronic acid moiety in the pentasaccharide is not mandatory for ATIII binding, and may be replaced by a 2-*O*-sulfated iduronic acid unit, as in synthetic octasaccharides which retain anticoagulant activity. Hence, sequences containing precursors of ΔIs and ΔIIIs, namely Is_id_ [IdoA(2S)-GlcN(NS,3S,6S)] and IIIs_id_ [IdoA(2S)-GlcN(NS,3S)], might bind to ATIII.

The selectivity of heparinase I in non-conventional 3S sequences can be used to augment the information obtained from building block analysis after heparin digestion by using mixtures such as heparinases II+III (30) or heparinase II alone (13) rather than all three heparinases. Since most heparin cleavage reactions can be obtained with heparinase II alone, differences between all three heparinases used together and heparinase II alone will be restricted to glycoserines and 3S disaccharides, respectively, arising as a result of the actions of heparinases III (31) and I. In heparinase II digests, 3S disaccharide units located on the reducing end (RE) cannot undergo any further NRS cleavage, and will therefore be depolymerized into longer oligosaccharides (tetra- or hexasaccharides). These differences will be highlighted in the second part of this study, which was carried out on low- and high-affinity fractions from BMH obtained by ATIII affinity chromatography. Building blocks will be tagged by sulfanilic acid (8) to enable detection of the non-reducing ends (NRE) and afford better resolution of building blocks.

In this study, the focus was intentionally on the 3S sequences observed in heparin. This was because one of the aims of this work was to improve the knowledge of biological functions of the heparin sequences resulting, after digestion, in the unsaturated 3S disaccharides. The specificities of heparinases, highlighted here, will then be used to obtain a fuller description of heparin 3S sequences. Alternatively, the method described can be applied to further elucidate the specificities of 3-*O*-sulfotransferase biosynthetic enzymes (23–26).

## Materials and Methods

### Materials

BMH was obtained from Opocrin (LDO Spa, Milano, Italy). All enzyme lyases from *Flavobacterium heparinum* [Heparinase I (EC 4.2.2.7), Heparinase II (no EC number), Heparinase III (EC 4.2.2.8)], Δ^4−5^ glycuronidase and Δ^4−5^ 2-O-sulfatase were obtained from Grampian Enzymes (Aberdeen). Water was purified using a Millipore Milli-Q purification system.

### Heparin Fractionation

#### ATIII Affinity Chromatography

An ATIII–Sepharose column (30 × 7 cm) prepared by coupling 2 g of human ATIII to CNBr-activated Sepharose 4B (Sigma) as described by Höök and coworkers (32) was used. A step gradient of NaCl concentration was applied. Low-affinity fractions were eluted using a 0.25 M NaCl solution buffered at pH 7.4 with 1 mM Tris–HCl at 11 mL/min; high-affinity fractions were eluted by a five-step gradient of NaCl (0.71, 1.39, 2.07, 2.72, and 3.5 M NaCl and 1 mM Tris–HCl, pH 7.4). The NaCl gradient was monitored by conductivity measurements, and heparin fractions were detected by ultraviolet (UV) light at 219 nm. This wavelength limits the influence of any NaCl present in the mobile phase and is sensitive to *N*-acetyl functional groups across the heparin chain. Chromatograms were obtained after subtraction of the signal obtained using a blank run. Injected quantities varied between 250 and 500 mg of heparin, depending on the capacity of the column and the type of heparin.

#### Desalting

Multistep desalting was necessary for the elimination of NaCl, especially for fractions of highest affinity. First, high-affinity fractions (1L) were diluted 1:5 in water and passed through a 20 × 1.6 cm column filled with Q-Sepharose Fast Flow (Sigma Aldrich, Saint-Quentin-Fallavier, France). The column was then washed with water to eliminate free NaCl. The heparin was then flash-eluted by NaClO_4_ 2.5 N. UV detection at 215 nm was used to monitor the elution. In a second step, the heparin solution was desalted on a 100 × 7 cm column filled with Sephadex G10 monitored with UV detection at 215 nm and conductimetry.

### Enzymatic Digestion

All heparinases were prepared at concentrations of 0.5 IU/mL in a pH 7.0 buffer [10 mM KH_2_PO_4_ and 0.2 mg/mL of bovine serum albumin (BSA)]. Depolymerizations with heparinase II and heparinases I+II+III were performed at room temperature for 48 h in a total volume of 170 μL containing 20 μL of a 20 mg/mL solution of heparin in water, 20 μL of a mixture of the heparinases at 0.5 IU/mL and 130 μL of 100 mM pH 7.0 buffer containing 2 mM Ca(OAc)_2_ and 0.5 mg/mL BSA.

### Reductive Amination With Sulfanilic Acid

Heparin building blocks generated by the digestion of heparins with heparinase were tagged by sulfanilic acid, as previously described (8). Oligosaccharides obtained after digestion were diluted to 200 μL with 4% acetic acid (v/v in water). They were introduced into a HPLC vial (1.7 mL) containing 4–6 mg of sulfanilic acid and 6–10 mg of picoline borane. The reaction was complete after 8 h at 37°C. The remaining reagents were removed on Sephadex G10 (column 30 × 2.6 cm) circulated with H_2_O/EtOH, 90/10 v/v.

### Chromatographic Analysis of Digests

#### Strong Anion Exchange (SAX) Chromatography on AS11 Column

Standard Carbopack AS11 conditions used a single 250 × 2.1 mm column (Thermo Scientific Dionex, Montigny-le-Bretonneux, France). The column temperature was set at 40°C. Mobile phase A was 2.5 mM NaH_2_PO_4_ at pH 2.8, and mobile phase B was an aqueous solution of 2.5 mM NaH_2_PO_4_ with 1 M NaClO_4_ adjusted to pH 3.0. A linear gradient (t_0min_ B% 0; t_80min_ B% 60) was applied for elution at a flow rate of 0.22 mL/min. Diode array detection was used. Double UV detection was performed at 232 nm and 202–247 nm.

#### Ion-Pair Liquid Chromatography/Mass Spectrometry

The liquid chromatography/mass spectrometry (LC/MS) method used to analyze heparin digests was as described previously (8), and used an Acquity UPLC BEH C18 column, 150 × 2.1 mm, 1.7 μm (Waters SAS, En Yvelines Cedex, France). Mobile phase A was water, and mobile phase B water:acetonitrile (30:70). The ion pairing reagent, heptyl amine (HPTA; 7.5 mM), and a buffering agent, hexafluroisopropanol (50 mM), were added to both A and B. A linear gradient (t_0min_ B% 1; t_70min_ B% 70) was applied for elution at a flow rate of 0.22 mL/min. Column temperature was set at 30°C and diode array detection used. Double UV detection was performed at 265 nm and 232 nm. Electrospray ionization mass spectra were obtained using a Waters Xevo Q-Tof mass spectrometer. The electrospray interface was set in negative ion mode with a capillary potential of 2000 V and a sampling cone potential of 20 V. Source and desolvation temperatures were 120 and 300°C, respectively. Nitrogen was used as the desolvation (750 L/min) and cone (25 L/min) gas. The mass range was 50–2500 Da (scan rate = 0.8 s). Acquisition was performed in MSE mode with low energy at 7 V and a high energy ramp from 30 to 50 V.

#### Reaction With Δ^4−5^ Glycuronidase and Δ^4−5^ 2-*O*-Sulfatase

The sulfanilic tagged heparin digests (100 μL) were diluted 1/3 to 1/5 in 5 mM Na_2_HPO_4_ at pH 7.0 before being treated for 24 h at room temperature with 20 milliunits of Δ^4−5^ 2-*O*-sulfatase or Δ^4−5^ glycuronidase.

## Results and Discussion

### Selectivity of Heparinase for 3-*O*-Sulfated Moieties

#### Selective Depolymerization of 3-*O*-Sulfated Disaccharides by Heparinase I

**Figure 1:**
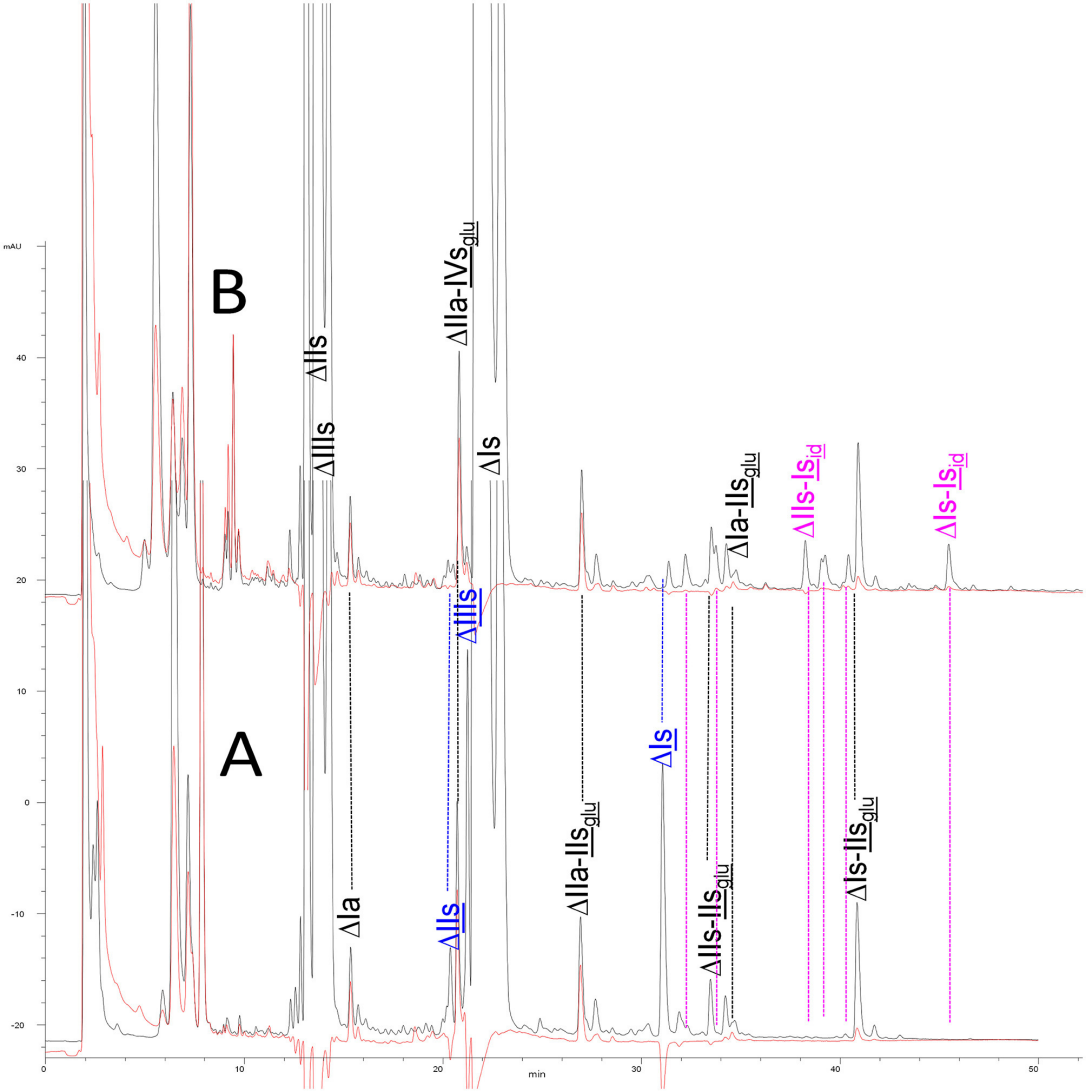
Comparison of digests from bovine mucosal heparin by **(A)** heparinase I+II+III, and **(B)** heparinase II, separated on a Carbopack AS11 column. Black line (**—**): UV 232 nm; red line (**—**): UV 202 nm-242 nm (UV signal specific to acetyl oligosaccharides).

To demonstrate that unsaturated 3S disaccharides are generated by heparinase I digestion, a BMH was digested with a heparinases I+II+III mixture and with heparinase II alone (Figure 1). In a preliminary statement, especially important when the selectivity of heparinase is concerned, it should be emphasized that heparinases from *Flavobacterium heparinum* were used in this work. We previously observed that heparinases obtained as recombinant enzymes may differ in activity level and specificity from their naturally generated counterparts, which could in turn influence the results of any equivalent analysis. In a similar way, heparinases from *Bacteroides eggerthii* (33) (New England Biolabs, Ipswich, MA) have demonstrated different substrate specificities compared with those from *Flavobacterium heparinum*.

Heparinase I preferentially cleaves highly sulfated zones and has a primary selectivity for GlcNS (± 6S)1 → 4IdoA(2S) linkages (20–22) with a pure endolitic mechanism (Supplementary Figure 1). By contrast, heparinase II has broader selectivity (20–22) but its mechanism of action is more complex. This enzyme has two separate actions with a high probability of the presence of two active sites (34), one acting endolitically and the other exolitically (Supplementary Figure 2). The processive exolytic mechanism is acting at the reducing end (unpublished results). However, the major characteristic of heparinase II in relation to this work is that it cannot cleave the non-reducing link of the 3S disaccharidic units (12, 14–17). In a more general way, despite its broad selectivity, the cleavage of low sulfated disaccharides such as ΔIVs (ΔHexUA-GlcNS) or even ΔIVa (ΔHexUA-GlcNAc) appears difficult, if not impossible, in sequences specifically cleaved by heparinase III. Heparinase III cleaves low-sulfated sequences, and therefore has no selective action on 3S moieties. The main heparinase III specificity is directed at the glycoserine bond ^↓^GlcA β1-3 Gal β1-3 Gal β1-4 Xyl β1- (31), and its absence from the lyase mixture is demonstrated by the presence in the digest of complete glycoserine moieties, with no effect on 3S building blocks. BMH is the only heparin in which ΔIIIs is present in significant amounts (8), and three unsaturated 3S disaccharides were accordingly detected in the chromatogram after heparinase I+II+III digestion. However, as shown in Figure 1 (chromatogram b), the depolymerization of 3S units into disaccharides is not possible using heparinase II since the NRS is no longer cleavable, with the digestion fragments eluting as higher oligosaccharides, where the 3S disaccharides are found at the RE, as in ΔIs-Is_id_ [(ΔHexUA(2S)-GlcN(NS,6S)-IdoA(2S)-GlcN(NS,3S,6S); see Table 2, lines 7 and 8] (Full structural identification in Supplementary Table 1).

**Table 2:** Comparison of heparinase II and heparinase I digestions of key oligosaccharides.

	**Oligosaccharide**	**Heparinase II**	**Heparinase I**
1	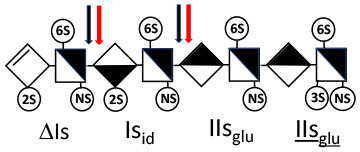	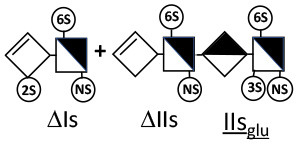	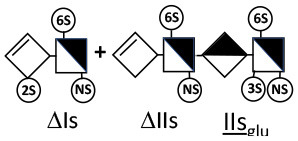
2	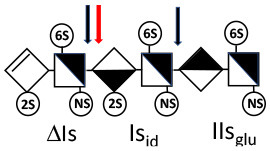	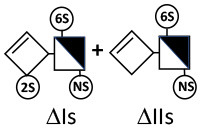	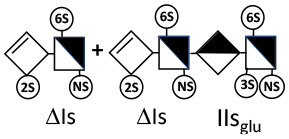
3	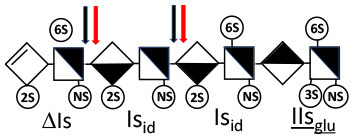	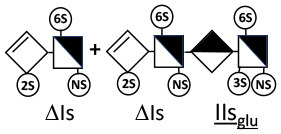	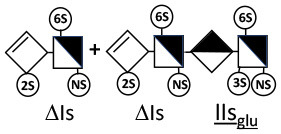
4	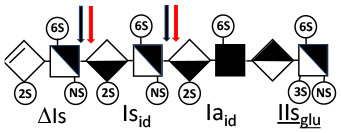	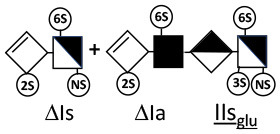	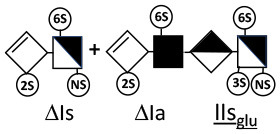
5	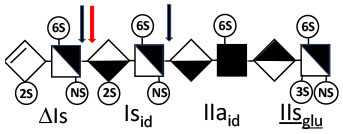	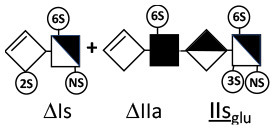	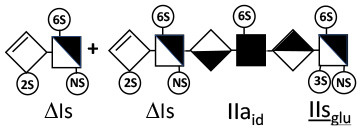
6	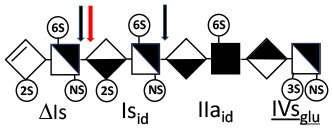	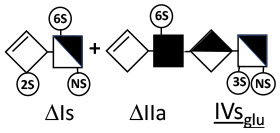	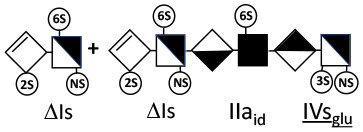
7	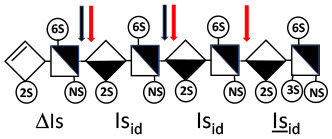	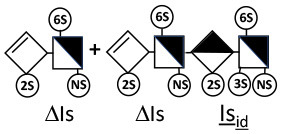	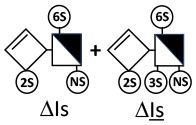
8	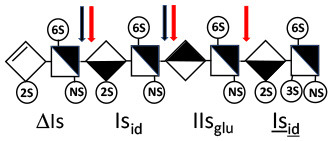	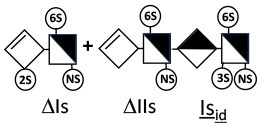	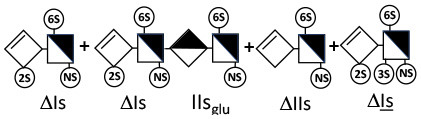
	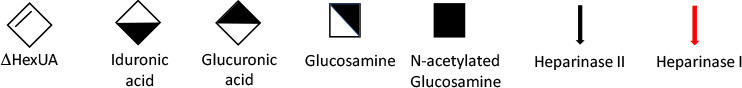		

**Figure 2:**
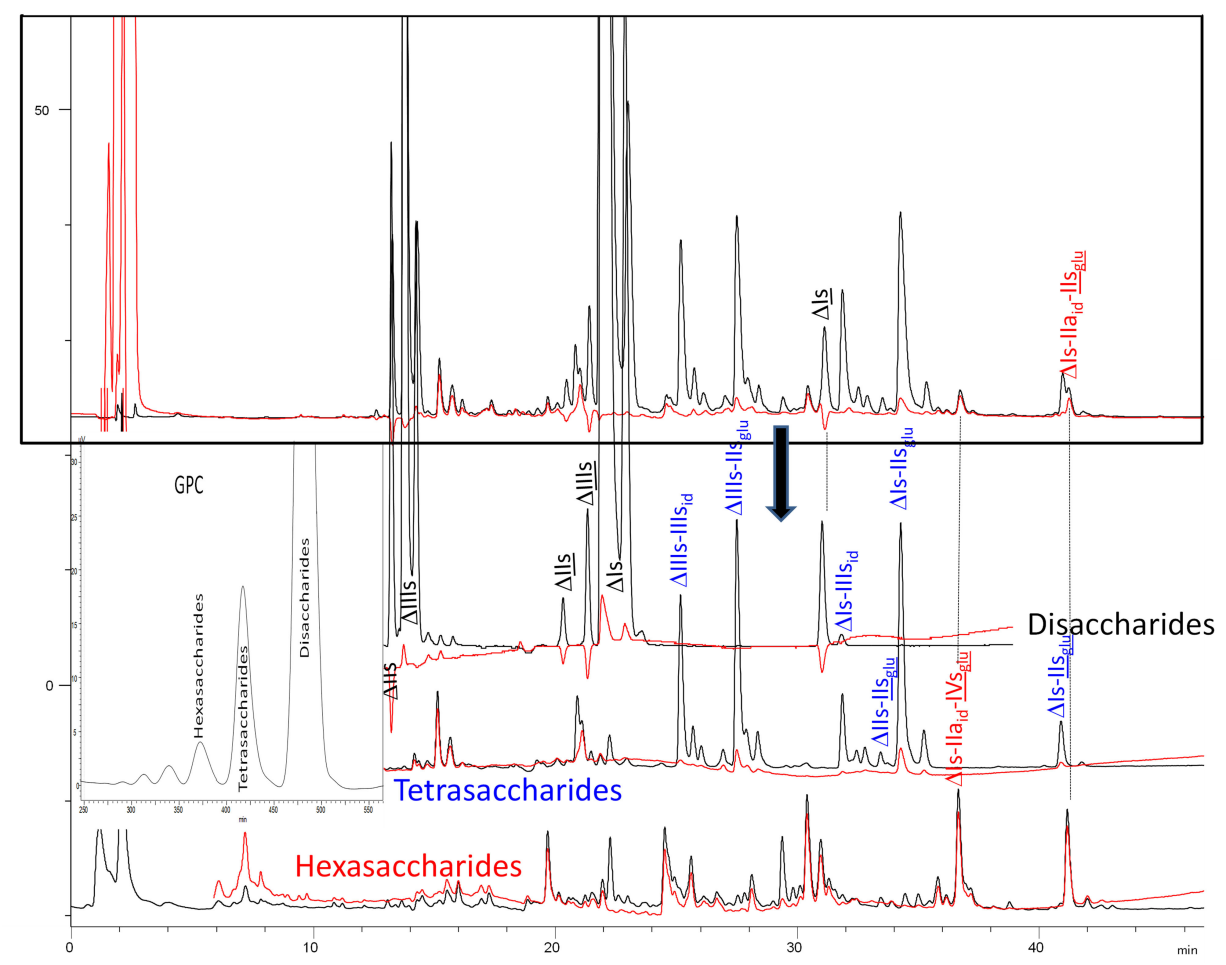
Heparinase I digest of bovine intestine heparin (BMH) separated on a Carbopack AS11 column. Disaccharides to hexasaccharides are the gel permeation chromatography fractions corresponding to the digest. Black line (**—**): UV 232 nm; red line (**—**): UV 202 nm-242 nm.

Chromatograms of the same heparin digested by heparinase I are shown in Figure 2. The digest is more complex than those shown in Figure 1, and gel permeation chromatography (GPC) was used to facilitate identification of components. In contrast to heparinase II, 3-*O*-sulfation facilitates rather than hinders cleavage on the non-reducing linkage. The three 3S disaccharides in Figure 2 are present at the same levels as found using mixed heparinases (Figure 1A), although only heparinase I was used. This is even the case for ΔIIs where in the absence of uronic 2-*O*-sulfate, the cleavage of the non-reducing linkage is considerably more difficult.

ΔIIs-IIs_glu_ [ΔHexUA-GlcN(NS,6S)-GlcA-GlcN(NS,3S,6S)] and ΔIs-IIs_glu_ [ΔHexUA(2S)-GlcN(NS,6S)-GlcA-GlcN(NS,3S,6S)] are also present (Figure 2). ΔIIs-IIs_glu_ appears as a result of cleavage of -$\text{Is}\frac{↓}{\text{id}}{\text{IIs}}_{\text{glu}}\text{-}{\underline{\text{IIs}}}_{\text{glu}}$, which predominates over -$\text{Is}\frac{↓}{\text{id}}{\text{IIs}}_{\text{id}}\text{-}{\underline{\text{IIs}}}_{\text{glu}}$ (note that the two hexasaccharides ΔIs-IIs_glu_-IIs_glu_ [(ΔHexUA(2S)-GlcN(NS,6S)-GlcA-GlcN(NS,6S))-GlcA-GlcN(NS,3S,6S)] and ΔIs-IIs_id_-IIs_glu_ [(ΔHexUA(2S)-GlcN(NS,6S)-IdoA-GlcN(NS,6S))-GlcA-GlcN(NS,3S,6S)] have been isolated from PMH after heparinase I digestion in our laboratory [unpublished results]). Given that ΔIs-IIs_glu_ [ΔHexUA(2S)-GlcN(NS,6S)-GlcA-GlcN(NS,6S)] (14) appears in the digest at reaction completion as a consequence of resistance to cleavage of the -$\text{Is}\frac{↓}{\text{id}}{\text{IIs}}_{\text{glu}}$ linkage (Table 2, lines 1 and 2), the generation by heparinase I of ΔIIs-IIs_glu_ is difficult to account for. The reason why -$\text{Is}\frac{↓}{\text{id}}{\text{IIs}}_{\text{glu}}\text{-}{\underline{\text{IIs}}}_{\text{glu}}$ is cleaved when -$\text{Is}\frac{↓}{\text{id}}{\text{IIs}}_{\text{glu}}$ is resistant can be explained by the novel observation of the distance of the link from the RE. In modeled reactions on key decasaccharides [ΔIs-Is_id_-Is_id_-Is_id_-Is_id_, ΔIs-Is_id_-Is_id_-Is_id_-IIIs_id_, ΔIs-Is_id_-Is_id_-Is_id_-IIs_glu_ (unpublished results)], progressive resistance to cleavage is observed as the bond moves closer to the RE.

The fact that resistance to cleavage can be increased approximately 4-fold when the link is directly on the RE is of major importance, especially when sequencing oligosaccharides purified from LMWH (35). Ernst et al. (19) highlighted this effect on key decasaccharides, but concluded incorrectly that the mechanism was exolytic. There is now an overall consensus across the literature (14, 36, 37) that the behavior of heparinase I is endolytic, as evident from GPC of heparin digests (14, 38). The influence of distance from the RE is essential for understanding the depolymerization mechanism of heparinase I, and for explaining why hexasaccharides like ΔIs-IIa_id_-IIs_glu_, are resistant, not because of, but despite, their 3-*O*-sulfation. In the heparinase I digests, prominent selectivity for cleavage of -GlcN(NS,3S,6S)^↓^IdoA(2S) linkages (14) is due to the highly sulfated environment. Under these conditions, endolytic cleavage first targets ATIII-binding pentasaccharides and generates 3S reducing glucosamines. For the main PMH binding site, -Is_id_-IIa_id_-IIs_glu_ [-IdoA(2S)-GlcN(NS,6S)-IdoA-GlcNAc(6S)-GlcA-GlcN(NS,3S,6S); Table 2, line 5], the ^↓^ IIs_glu_ (^↓^GlcA-GlcN(NS,3S,6S)) link cannot be cleaved because of (i) its position on the RE and (ii) low sulfation directly on both sides of the non-reducing link. Furthermore, the absence of 2-*O*-sulfation, and importantly the *N*-acetylation of the glucosamine moiety, inhibits -$\text{Is}\frac{↓}{\text{id}}{\text{IIs}}_{\text{id}}\text{-}{\underline{\text{IIs}}}_{\text{glu}}$ cleavage so that the hexasaccharide ΔIs-IIa_id_-IIs_glu_ is obtained (15). As indicated in Figure 2, when the glucosamine is *N*-sulfated, cleavage is possible, and a tetrasaccharide is obtained (case of ΔIIs-IIs_glu_ and ΔIs-IIs_glu_). For *N*-acetylated pentasaccharides, the cleavage of the NRS link is possible only where 2-*O*-sulfation is present, as in -Is_id_-Ia_id_-IIs_glu_ (-IdoA(2S)-GlcN(NS,6S)-IdoA(2S)-GlcNAc(6S)-GlcA-GlcN(NS,3S,6S), yielding the tetrasaccharide ΔIa-IIs_glu_ (Table 2, lines 1 and 4). In all these cases, 3-*O*-sulfation triggers only the first cleavage, which places the 3S glucosamine at the RE. Note that cleavage selectivity of heparinase I for this new sequence, containing the 3S glucosamine on the RE, is no longer dependent on the reducing 3-*O*-sulfate, and identical cleavage would occur on a similar sequence without any 3S.

In the digestion by heparinase I of key decasaccharides, cleavage of intermediate tetrasaccharides (ΔIs-Is_id_ for ΔIs-Is_id_-Is_id_-Is_id_-Is_id_) (ΔIs-Is_id_ and ΔIs-IIIs_id_ for ΔIs-Is_id_-Is_id_-Is_id_-IIIs_id_) starts only after the elimination of all other longer fragments as a result of decreasing lyase activity at the RE. In general, tetrasaccharide digestion by heparinase I is possible only when sulfation in positions 2, 6 and *N* is present. If the glucosamine is 6-OH, as in ΔIs-IIIs_id_, digestion is very difficult. It is also blocked when the uronic acid is 2-OH, especially with glucuronic configurations as in ΔIs-IIs_glu_. Therefore, the tetrasaccharide residues of ATIII-binding sites, even the most heavily sulfated (ΔIs-IIs_glu_), cannot be reduced to disaccharides (or to only a very small extent). The only exception is when the unsaturated disaccharide is 3S as in ΔIIs-IIs_glu_ [ΔHexUA-GlcN(NS,3S,6S)-GlcA-GlcN(NS,3S,6S)], which is at least partially digested into ΔIIs depending on the quantity of heparinase I added (Supplementary Figure 6).

#### Generation of Unsaturated 3-*O*-Sulfated Disaccharides by Heparinases

The presence in heparinase I digests of the 3S disaccharides ΔIs, ΔIIs and ΔIIIs (Figure 2) raises the question of the structural parameters which allow complete digestion to these products. In the case of ΔIs and ΔIIIs, 2-*O*-sulfation of the uronic acid is the key factor: when associated with 3-*O*-sulfation of the glucosamine residue, NRS cleavage is observed whatever the configuration of the uronic acid. The question of the presence of ΔIIs in heparin digests is less straightforward. If we consider results obtained with synthetic 3S hexasaccharides (18), unsaturated 3S disaccharides would always be obtained when the glucosamine on the NRS of the 3S unit is N- and 6-*O*-sulfated, since the cleavage by heparinase I is possible in all proposed examples of that type. However, these observations cannot be directly applied to 3S sequences in heparin chains because the position of the 3S glucosamines in this work are not on the RE, as in digested heparin. Instead, the 3S glucosamines are in the medium position of the hexasaccharides, on the NRS of the reducing disaccharide, which induces specific heparinase I behavior.

Similarly, in sequencing experiments on oligosaccharides purified from high-affinity fractions of LMWH [enoxaparin (39) and semuloparin (40)] carried out at our laboratory, the formation of ΔIIs from -IIs_glu_- was frequently observed when the glucosamine on the NRS was N-sulfated. These experiments started with a short oligosaccharide (hexasaccharide to decasaccharide) containing the entire ATIII binding site. Consequently, the cleavage on -IIs_glu_^↓^ by heparinase I was frequently hindered by simple inhibition at the reducing link or by another reducing moiety resistant to heparinase I such as a mannosamine or a 1,6-anhydro ring (39). Under these circumstances, heparinase I first cleaved the non-reducing link^↓^ IIs_glu_-, resulting ultimately in the formation of ΔIIs.

This behavior is due to specific structures generated by the LMWH depolymerization process, but these structural features could not be found (or were present only in trace amounts) in a heparin partially digested by heparinases. In such cases, the 3S glucosamines are predominantly situated at REs after preliminary cleavage (as described previously), and heparinase-resistant 3S tetrasaccharides are obtained. When the specificity of heparinase I is considered, it is difficult to rule out possible switching to an iduronic configuration, i.e., -IIs_id_ [-IdoA-GlcN(NS,3S,6S)], which could probably allow ΔIIs transformation, or at least facilitate it. This has been reported previously by Mochizuki et al. (17). IIs_id_ could be obtained from IIs_id_ with the presence of 3-*O*-sulfotransferases (3-OST) (24, 41): ΔIIs has been detected (23, 26) in 3-OST-modified heparan sulfate. Thus, the possibility to obtain ΔIIs from IIs_id_ must be examined.

However, several factors militate against the IIs_id_ hypothesis: first, ΔIIs is absent in ATIII low-affinity heparin fractions, and increases with affinity of the heparin fraction for ATIII (see later). Therefore, ΔIIs cannot be classified as a non-ATIII binding 3-*O*-sulfate, as in Mochizuki et al. (17). Furthermore, it appears that IIs_id_ is not compatible with the structural requirements of ATIII binding sequences and sequences containing IIs_id_ have never been found to bind ATIII (18). Additionally, in nitrous depolymerizations of heparins, the transformed building block of IIs_id_, IdoA-aMan3S6S (in which aMan represents 2,5-anhydromannose), has never been detected (42). Indeed, we believe that ΔIIs results from digestion by heparinase I of sequences with consecutive 3-*O*-sulfated units.

In the next section, where digestion by heparinase mixture and by heparinase II alone are compared, ATIII binding sites with a supplementary 3-*O*-sulfate such as -IIa_id_-IIs_glu_-Is_id_- [IdoA-GlcNAc(6S)-GlcA-GlcN(NS,3S,6S)-IdoA(2S)-GlcN(NS,3S,6S)] are found in heparin fractions with highest affinity for ATIII. They are detected in heparinase II digests as unsaturated hexasaccharides ΔIIa-IIs_glu_-Is_id_. ΔIIa-IIs_glu_-Is_id_-Is_id_ was identified (43) in our laboratory in structural studies on the high ATIII affinity octasaccharide fraction from semuloparin, which contains this unusual 3S element. Furthermore, the same sequence had already been reported in ATIII high-affinity fractions of BMH (27). Based on this accumulating evidence, we believe that heparin contains other types of consecutive 3S units, including -IIs_glu_-. Several examples have been isolated in ATIII high-affinity fractions of PMH in our laboratory. The full structural identification of one of these, the decasaccharide ΔIs-IIa_id_-IIs_glu_-IIs_glu_-IIs_glu_ [ΔHexUA(2S)-GlcN(NS,6S)-IdoA-GlcNAc(6S)-GlcA-GlcN(NS,3S,6S)-GlcA-GlcN(NS,3S,6S)-GlcA-GlcN(NS,3S,6S), is shown in Supplementary Table 2, followed by its sequencing via fractionation with heparinase in Supplementary Figures 6, 7]. In the presence of heparinase II, a mixture of ΔIIa-IIs_glu_-IIs_glu_-IIs_glu_ and ΔIs is obtained. Digestion by heparinase I first gives a mixture of ΔIs-IIa_id_-IIs_glu_, ΔIIs-IIs_glu_ and ΔIIs, and then ΔIs-IIa_id_-IIs_glu_ and ΔIIs only at completion.

### Analysis of BMH Fractions With Various Affinities for ATIII: Exhaustive vs. Specific Heparinase II Digests

**Figure 3:**
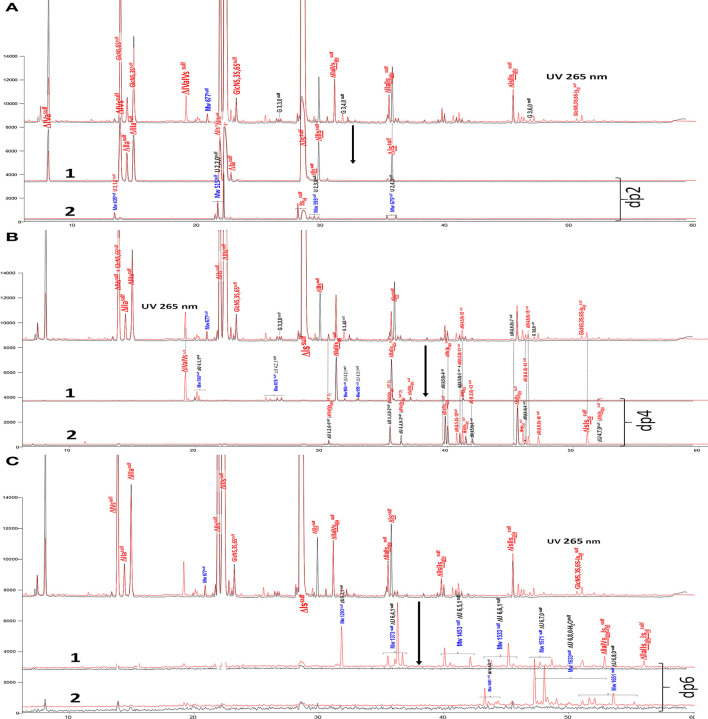
**(A)** Comparison by liquid chromatography/mass spectrometry ion pair chromatography of heparinase I+II+III (**—**) and heparinase II (**—**) digests of bovine mucosal heparin after sulfanilic reductive amination. Reconstructed ion pair chromatograms (RIC) of disaccharide (dp2 are compared to UV chromatograms detected at 265 nm. (**1**) RIC unsaturated disaccharides: m/z 535.14+573.07+615.094_615.2+652.906 _653.05+695+848.1+1043.2; (**2**) RIC non-reducing end disaccharides: m/z 591.1+671.1+866.2+1061.3 (Correspondence of m/z values is specified in Supplementary Table 3). **(B)** Comparison by liquid chromatography/mass spectrometry ion pair chromatography of heparinase I+II+III (**—**) and heparinase II (**—**) digests of bovine mucosal heparin after sulfanilic reductive amination. Reconstructed ion pair chromatograms (RIC) of tetrasaccharide (dp4) are compared to UV chromatograms detected at 265 nm. (**1**) RIC acetylated tetrasaccharides: m/z 555.6+595.6+635.5+515.6+952.2; (**2**) RIC sulfated tetrasaccharides: m/z 614.5+712 (Correspondence of m/z values is specified in Supplementary Table 3). **(C)** Comparison by liquid chromatography/mass spectrometry ion pair chromatography of heparinase I+II+III (**—**) and heparinase II (**—**) digests of bovine mucosal heparin after sulfanilic reductive amination. Reconstructed ion pair chromatograms (RIC) of (**1**) RIC acetylated hexasaccharides: m/z 724.156_724.3+764.1+861.7+959.2+1056.8 +1154.3; (**2**) RIC sulfated hexasaccharides: m/z 880.6+978.2+1133.3 (Correspondence of m/z values is specified in Supplementary Table 3).

BMH is the most interesting heparin with which to illustrate differences between heparinases I+II+III and heparinase II digests, because the analytical gap between the two digests is maximized in this specie in large part due to high content of unsaturated 3S disaccharides. Their absence in the heparinase II digest is confirmed in the reconstructed ion pair chromatograms (RICs) of disaccharides in Figure 3A-1. Within disaccharides, ΔIVa and, to a lesser extent, ΔIVs are less prevalent in the heparinase II digests, basically because of the absence of heparinase III. On the full LC/MS ion pair chromatogram (8) of the heparinase II digests of this BMH batch (Supplementary Figure 8), the glycoserine building blocks present in the exhaustive heparinases I+II+III digest were absent when heparinase II only was used, and eluted as higher oligosaccharides where the glycoserine is preceded on the NRS by one or two low-sulfated disaccharides such as IVa, IIa or IVs. For NRE disaccharides (Figure 3A-2), the two digests are identical, as expected, because in these building blocks, the absence of NRS links structurally eliminates any possible selective cleavage on that side by heparinases.

Tetrasaccharide building blocks were shared between acetylated (Figure 3B-1) and non-acetylated species (Figure 3B-2). For acetylated tetrasaccharides, the main difference is for ΔIVa-IVs because of selective cleavage by heparinase III. The RIC of sulfated tetrasaccharides in Figure 3B-2 reveals the unique structural diversity of BMH due to its low 6-*O*-sulfation (6-OH 38%). For ΔU(4,5,0) tetrasaccharides, five isomers other than ΔIIs-IIs_glu_ were detected in the heparinase I+II+III digest (ΔU(4,5,0)-4, ΔU(4,5,0)-5, ΔU(4,5,0)-6, ΔIIIs-IIs_glu_ and ΔIs-IVs_glu_). Moreover, in the heparinase II digest, four other new ΔU(4,5,0) tetrasaccharides were detected, corresponding to the building blocks derived from 3S disaccharides in the absence of heparinase I. In the higher sulfation range, four ΔU(4,6,0) and two ΔU(4,7,0) (one minor) new tetrasaccharides were present in the heparinase II digest. Only two of them were previously known: ΔIs-Is_id_ (isolated from a BLH low ATIII affinity fraction digested by heparinase I, see Supplementary Table 1), and ΔIIs-Is_id_, identified by the action of Δ^4−5^ 2-*O*-sulfatase on ΔIs-Is_id_. These two tetrasaccharides were also detected in lower amounts in other heparin sources. The structures of the tetrasaccharides detected in Figure 3B-2 will be discussed later.

Figure 3C-1,2 also show RICs of hexasaccharides separated according to acetylation. Hexasaccharides are only present in trace amounts in the exhaustive digest. However, in the heparinase II digest, a small but significant hexasaccharide fraction was observed. Some [ΔU(6,3,1), ΔU(6,4,1)] may be attributable to the absence of heparinase III, while others show resistance to digestion which is likely to be due to the presence of two consecutive 3S disaccharides. Here, only two (ΔIIa-IIs_glu_-Is_id_ and ΔIIa-IVs_glu_-Is_id_) have been identified.

BMH fractions isolated from ATIII affinity chromatographs were used to study these new building blocks further.

#### Affinity Chromatography on Immobilized ATIII

**Figure 4:**
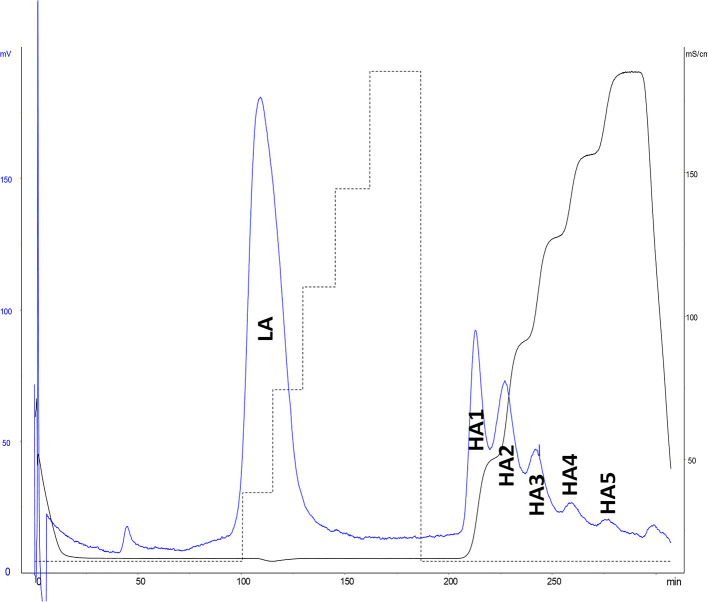
ATIII affinity chromatography of bovine mucosal heparin (BMH). Detection by UV at 219 nm and conductimetry.

ATIII affinity chromatography was used to fractionate heparins into an ATIII low-affinity fraction (LA) and five fractions of increasing affinity for ATIII (HA1 to HA5) (Figure 4). A five-step concentration gradient (0.71, 1.39, 2.07, 2.72, 3.5 M NaCl) with injection of ~500 mg of heparin was used. The properties of BMH and collected fractions are summarized in Table 3.

**Table 3:** Characteristics of bovine mucosal heparin (BMH, starting heparin) and observed ATIII affinity chromatography fractions, with proportions.

	**Quantity (g)**	**Proportion (%)**	**Molecular weight (Da)**	**AXa (IU/mg)**	**AIIa (IU/mg)**
**BMH**			**17,100**	**133**	**136**
**LA**	14.2	63.9	15,100	NA	NA
**HA1**	1.63	7.3	19,800	NA	NA
**HA2**	2.56	11.5	19,600	NA	NA
**HA3**	2.14	9.7	18,300	NA	NA
**HA4**	1.17	5.3	20,300	NA	NA
**HA5**	0.50	2.3	18,500	NA	NA

*NA: not applicable*.

**Figure 5:**
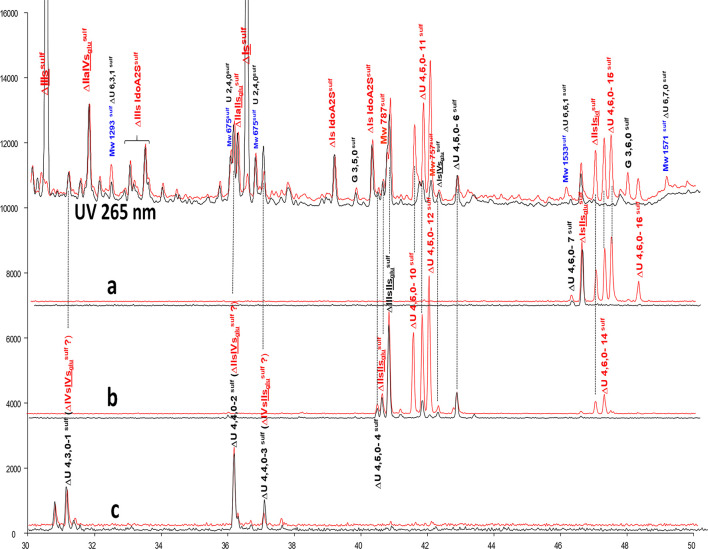
Comparison by ion-pair liquid chromatography/mass spectrometry of heparinase I+II+III (**—**) and heparinase II (**—**) digests of bovine mucosal heparin low-affinity (LA) fraction after sulfanilic reductive amination. **(a)** Reconstructed ion pair chromatograms (RIC) ΔU(4,6,0)^sulf^: m/z 712.2; **(b)** RIC ΔU(4,5,0)^sulf^: m/z 614.5+672.1; **(c)** RIC ΔU(4,4,0)^sulf^+ ΔU(4,3,0)^sulf^: m/z 534.5.

**Figure 6:**
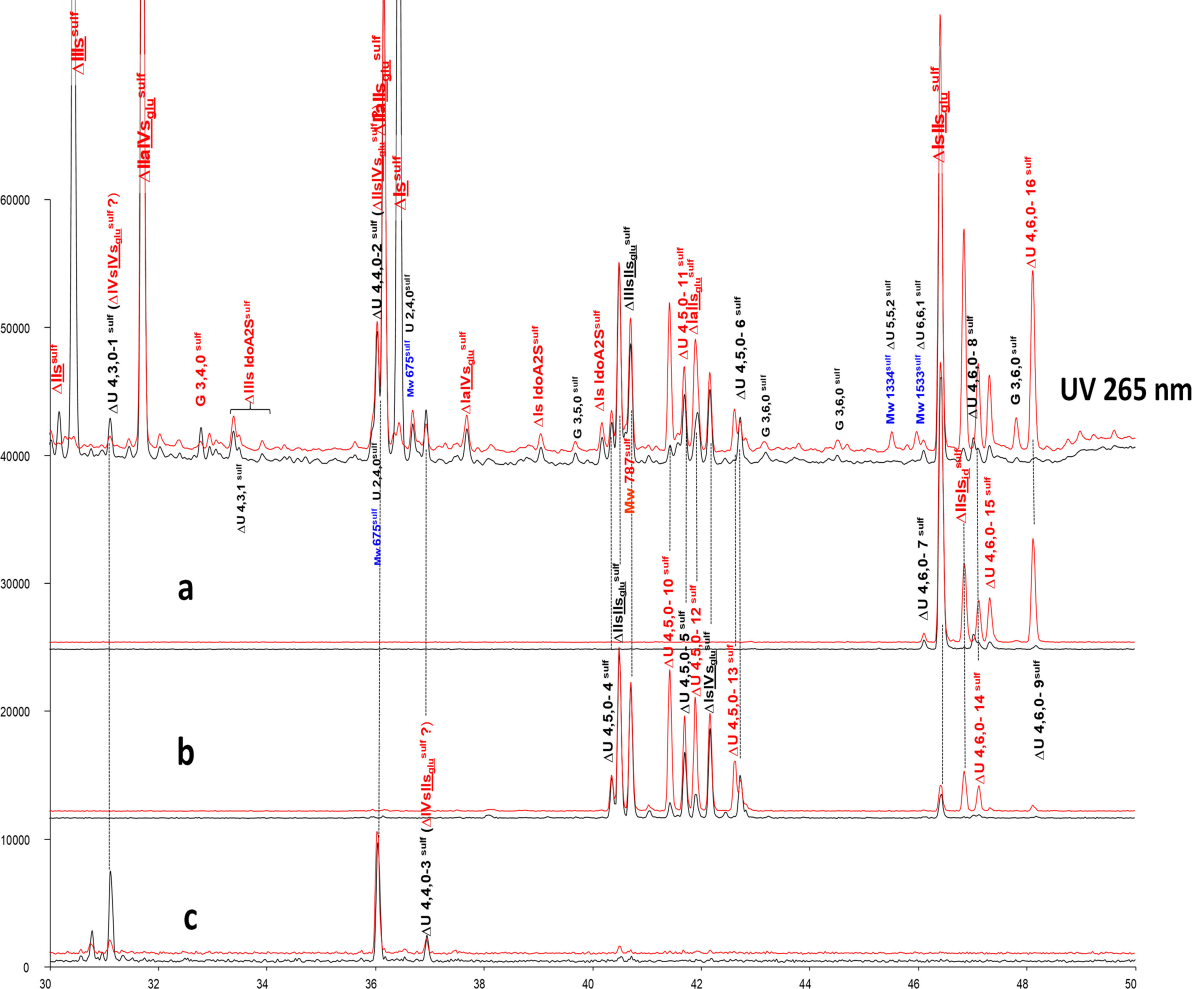
Comparison by ion-pair liquid chromatography/mass spectrometry of heparinase I+II+III (**—**) and heparinase II (**—**) digests of bovine mucosal heparin (BMH) high-affinity (HA3) fraction after sulfanilic reductive amination. **(a)** Reconstructed ion pair chromatograms (RIC) ΔU(4,6,0)^sulf^: m/z 712.2; **(b)** RIC ΔU(4,5,0)^sulf^: m/z 614.5+672.1; **(c)** RIC ΔU(4,4,0)^sulf^+ ΔU(4,3,0)^sulf^: m/z 534.5.

**Figure 7:**
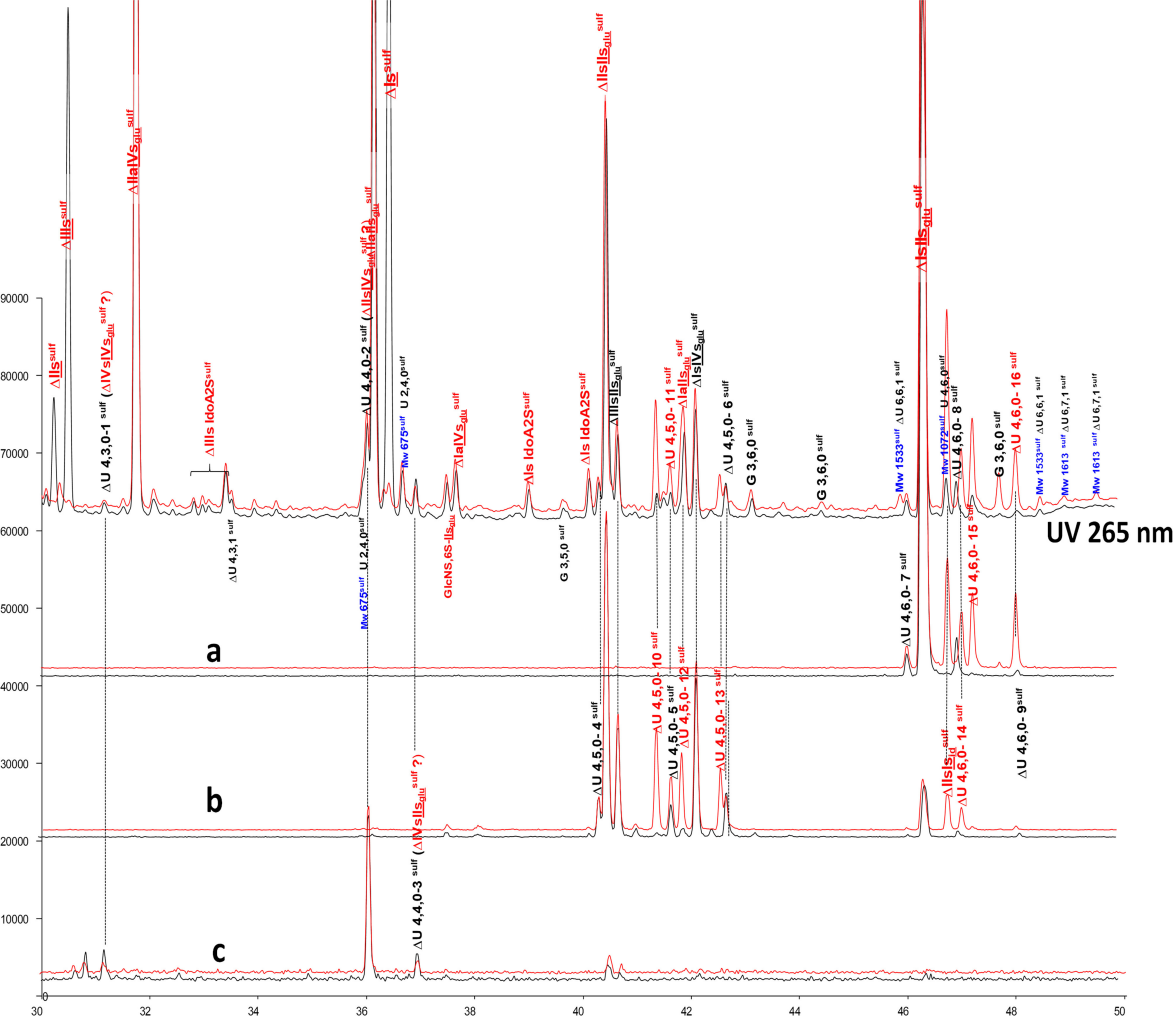
Comparison by ion-pair liquid chromatography/mass spectrometry of heparinase I+II+III (**—**) and heparinase II (**—**) digests of bovine mucosal heparin high-affinity (HA1) fraction after sulfanilic reductive amination. **(a)** Reconstructed ion pair chromatograms (RIC) ΔU(4,6,0)^sulf^: m/z 712.2; **(b)** RIC ΔU(4,5,0)^sulf^: m/z 614.5+672.1; **(c)** RIC ΔU(4,4,0)^sulf^+ ΔU(4,3,0)^sulf^: m/z 534.5.

**Figure 8:**
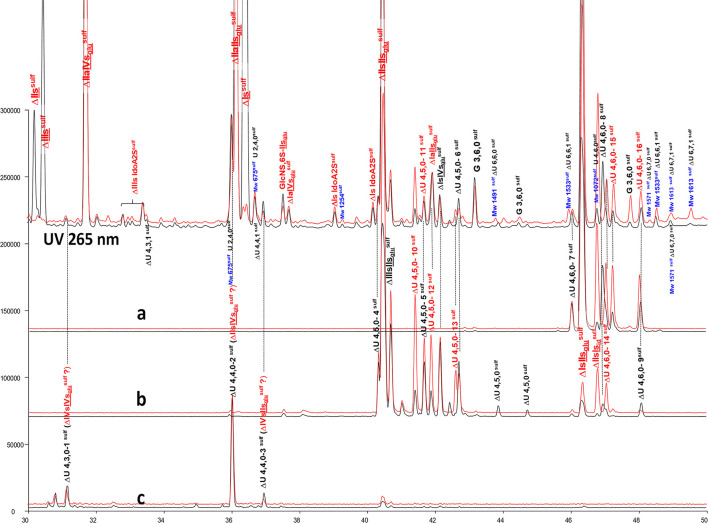
Comparison by ion-pair liquid chromatography/mass spectrometry of heparinase I+II+III (**—**) and heparinase II (**—**) digests of bovine mucosal heparin high-affinity (HA5) fraction after sulfanilic reductive amination. **(a)** Reconstructed ion pair chromatograms (RIC) ΔU(4,6,0)^sulf^: m/z 712.2; **(b)** RIC ΔU(4,5,0)^sulf^: m/z 614.5+672.1; **(c)** RIC ΔU(4,4,0)^sulf^+ ΔU(4,3,0)^sulf^: m/z 534.5.

Unidentified building blocks were detected in fractions from BMH (same batch as analyzed in Figure 3). The main tetrasaccharides in Figure 3B-2 (other than the five basic 3S tetrasaccharides ΔIIa-IIs_glu_, ΔIIa-IVs_glu_, ΔIIs-IIs_glu_, ΔIa-IIs_glu_, ΔIs-IIs_glu_) correspond to one ΔU(4,3,0) (peak 1), two ΔU(4,4,0) (peaks 2 and 3), five ΔU(4,5,0) (peaks 4 to 6, ΔIIIs-IIs_glu_ (ΔHexUA(2S)-GlcNS-GlcA-GlcN(NS,3S,6S), ΔIs-IVs_glu_ [ΔHexUA(2S)-GlcN(NS,6S)-GlcA-GlcN(NS,3S)] and three ΔU(4,6,0) (peaks 7 to 9). The LC/MS ion pair chromatogram digests (heparinase I+II+III and II alone) of LA, HA1, HA3 and HA5 from BMH with RIC corresponding to ΔU(4,3,0), ΔU(4,4,0), ΔU(4,5,0) and ΔU(4,6,0) are shown in Figures 5–8. Peaks ΔU(4,5,0)-4, ΔU(4,6,0)-7, ΔU(4,6,0)-8 and ΔU(4,6,0)-9 are not specific to BMH, and were also present in BLH digests. The remainder appear to be linked to 6-*O*-desulfation, so that their content (estimated with UV at 265 nm using LC/MS ion pair chromatography) is much lower in heparin sources other than BMH (Table 4). Δ^4−5^ glycuronidase and Δ^4−5^ 2-*O* sulfatase were applied to all digests to limit possible search options, and their action on each tetrasaccharide is also included in Table 4.

**Table 4:** Quantification (± 10%) of undetermined tetrasaccharide building blocks (% w/w) for bovine mucosal heparin (BMH; starting heparin) affinity fractions using ion-pair liquid chromatography/mass spectrometry (LC/MS).

	**ΔU(4,3,0)**	**ΔU(4,4,0)**		**ΔU(4,5,0)**						**ΔU(4,6,0)**			
	**1**	**2^a^**	**3**	**4**	**ΔIIs-IIs**_**glu**_ ^**a**^	**ΔIIIs-IIs**_**glu**_ ^**b**^	**5^b^**	**ΔIs-IVs**_**glu**_ ^**b**^	**6**	**7^b^**	**ΔIs-IIs**_**glu**_ ^**b**^	**8**	**9**

^*^	**ΔIVs-IVs**_**glu**_	**ΔIIs-IVs**_**glu**_	**ΔIVs-IIs**_**glu**_									**ΔIIs** **-** IIs_**glu**_	
**BMH**	**–**	**0.17**	**0.12**	**0.06**	**0.37**	**0.22**	**0.13**	**0.10**	**0.09**	**0.03**	**0.93**	**0.04**	**0.01**
**LA**	–	0.16	0.12	0.05	0.05	0.19	0.06	0.04	0.08	0.01	0.08	–	–
**HA1**	0.01	0.29	0.13	0.11	0.52	0.33	0.19	0.20	0.14	0.05	1.19	0.08	0.06
**HA2**	0.01	0.19	0.10	0.08	0.66	0.26	0.08	0.20	0.10	0.04	1.84	0.07	0.05
**HA3**	0.01	0.17	0.09	0.08	1.04	0.21	0.07	0.27	0.09	0.06	3.65	0.12	0.05
**HA4**	0.02	0.30	0.11	0.11	2.15	0.24	0.10	0.20	0.12	0.09	3.10	0.30	0.09
**HA5**	0.01	0.44	0.12	0.13	2.65	0.27	0.14	0.17	0.16	0.11	2.37	0.47	0.15

*Reference values: ΔIIs-IIs_glu_ and ΔIs-IIs_glu_ from AS11 building block analysis (8)*.

a *Digested by Δ^4−5^ glycuronidase*.

b *Digested by Δ^4−5^ 2-O-sulfatase*.

* *Hypothesis of structure*.

The constituents of exhaustive digests from BMH ATIII affinity fractions have already been described by Naggi et al. (27, 44). Four supplementary 3S tetrasaccharide building blocks (two with molecular weight Mw 994 Da [ΔU(4,4,0)] and two at 1074 Da [ΔU(4,5,0)] were detected in this work. Structures tentatively proposed were ΔIIs-IVs_glu_ [ΔHexUA-GlcN(NS,6S)-GlcA-GlcN(NS,3S)] and ΔIIIs-IVs_glu_ [ΔHexUA(2S)-GlcNS-GlcA-GlcN(NS,3S)] for the two at 994 Da, and ΔIIIs-IIs_glu_ and ΔIs-IVs_glu_ for those at 1074 Da. In accordance with Naggi et al. (27, 44), ΔIIIs-IIs_glu_ and ΔIs-IVs_glu_ appear to be appropriate choices for two of the five ΔU(4,5,0) unknowns in light of their distribution in the affinity fractions and their transformation by Δ^4−5^ glycuronidase and Δ^4−5^ 2-*O*-sulfatase.

Among the tetrasaccharides listed in Table 4, five were sensitive to Δ^4−5^ 2-*O*-sulfatase: the two tetrasaccharides identified as ΔIIIs-IIs_glu_ and ΔIs-IVs_glu_, ΔIs-IIs_glu_, and ΔU(4,5,0)-5 and ΔU(4,6,0)-7. The 6-*O*-sulfate on the non-reducing glucosamine of the ATIII pentasaccharide binding site is a major contributor to the affinity for ATIII (45). It corresponds to the one in the unsaturated unit of the tetrasaccharides, so that the presence in LA fractions of ΔIIIs-IIs_glu_ as a major ΔU(4,5,0) component is easily explained by the 6-*O*-desulfation of ΔIIIs-. Low amounts (<0.1%) of the ΔU(4,3,0) tetrasaccharide ΔIVs-IVs_glu_ [ΔHexUA-GlcNS-GlcA-GlcN(NS,3S)] (initially present in trace amounts) were also obtained when Δ^4−5^ 2-*O*-sulfatase was added. This was due to digestion of ΔIIIs-IVs_glu_ as proposed in (44) as a possible ΔU(4,4,0). However, additionally, peaks ΔU(4,4,0)-2 and ΔU(4,4,0)-3 increased as a result of the enzymatic Δ^4−5^ 2-*O*-desulfation of ΔIIIs-IIs_glu_ and ΔIs-IVs_glu_. It thus seems logical to propose for these two peaks the structures ΔIVs-IIs_glu_ [ΔHexUA-GlcNS-GlcA-GlcN(NS,3S,6S)] and ΔIIs-IVs_glu_ with a coelution (probably for peak ΔU(4,4,0)-2) reflecting the additional presence of ΔIIIs-IVs_glu_.

Among the species shown in Table 4, only ΔIIs-IIs_glu_ was fully digested by Δ^4−5^ glycuronidase, while a partial sensitivity was observed for tetrasaccharide ΔU(4,4,0)-2, with the concomitant formation of a trisaccharide, G(3,4,0) (Mw 836Da). As Δ^4−5^ glycuronidase has stronger reactivity on 6-*O*-sulfated substrates (46), peak ΔU(4,4,0)-2 could be due to ΔIIs-IVs_glu_, and then, consequently, peak ΔU(4,4,0)-3 due to ΔIVs-IIs_glu_. In contrast to hexasaccharide building blocks, complete or partial inhibition of Δ^4−5^ glycuronidase was frequently observed in the tetrasaccharides. Absence of reaction to Δ^4−5^ 2-*O*-sulfatase thus appears a better indicator of a Δ2-OH uronic acid than sensitivity to Δ^4−5^ glycuronidase. Another simple way to check Δ2-*O*-sulfatation of a component can be found in the chromatographs of the digests before sulfanilic tagging as shown in Figures 1, 2 through its UV spectrum by the 2 nm hypsochromic shift in the 232 nm maximum of UV absorbance in the case of a 2-*O*-sulfated unsaturated acid (6). The presence of a Δ2-OH uronic acid was thus ensured for tetrasaccharides ΔU(4,5,0)-4, ΔU(4,6,0)-8 and ΔU(4,6,0)-9, in line with the absence of reaction to Δ^4−5^ 2-*O*-sulfatase. The action of Δ^4−5^ 2-*O*-sulfatase on a digest of BLH fraction HA1 was used to confirm that tetrasaccharide ΔU(4,5,0)-4 is Δ2-*O*-desulfated tetrasaccharide ΔU(4,6,0)-7, as per the tetrasaccharides ΔU(4,4,0)-2 and ΔU(4,4,0)-3 corresponding to the Δ2-*O*-desulfated tetrasaccharides ΔIs-IVs_glu_ and ΔIIIs-IIs_glu_, respectively.

Tetrasaccharides ΔU(4,6,0)-8 and ΔU(4,6,0)-9 are however a special case, in that they are detected preferentially in HA4 and HA5 fractions. In addition, when digestion is limited to heparinase II, they disappear and are shifted to hexasaccharides as ΔIIs and ΔIIIs are to tetrasaccharides. Thus, the corresponding unsaturated disaccharide should also be 3-*O*-sulfated, and the heparinase II-corresponding hexasaccharide building blocks ΔU(6,8,0), ΔU(6,9,0) or ΔU(6,7,1) in the case of *N*-acetylation. ΔIIs-IIs_glu_ appears to be an ideal candidate, even if the full digestion of ΔIIs-IIs_glu_ by heparinase I was observed in the sequencing experiment of ΔIs-IIa_id_-IIs_glu_-IIs_glu_-IIs_glu_ (Supplementary Figure 6). The good correlation observed between the percentages of ΔU(4,6,0)-8 and ΔU(4,6,0)-9 with the percentage of ΔIIs, in BMH and BLH, argues in favor of their partial depolymerization into ΔIIs with some ΔIIs-IIs_glu_ remaining and corresponding to ΔU(4,6,0)-8. It should be noted that two ΔU(4,6,0) tetrasaccharides other than ΔIs-IIs_glu_ have already been reported in BLH (47).

#### Heparinase II Digestion of Heparin Affinity Fractions

##### Unknown Tetrasaccharide Building Blocks

Figure 3 shows that heparinase II-specific building blocks in BMH digests may be divided essentially into tetra- and hexasaccharides. It is interesting to note that all tetrasaccharides are *N*-sulfated, while hexasaccharides are the minimum possible for *N*-acetylated sequences. Additionally, there are two NRE trisaccharides, detected in BMH ATIII HA fractions. One of these, GlcN(NS,3S,6S)-Is_id_ [GlcN(NS,3S,6S)-IdoA(2S)-GlcN(NS,3S,6S)] (Supplementary Figure 8) has been identified previously in heparinase II digests of high-affinity hexasaccharides from enoxaparin; the second, G(3,6,0) (tr 47.3 min), is a mono-desulfated derivative of GlcN(NS,3S,6S)-Is_id_, possibly in position 6-*O*- of the NRE glucosamine. The same derivatives were also detected in BLH HA fractions. Interstingly, GlcN(NS,3S,6S)-Is_id_ is present in lower amount in PMH heparinase II digests, but in contrast, in this later case, the tetrasaccharide IIs_glu_-Is_id_ [GlcA-GlcN(NS,3S,6S)-IdoA(2S)-GlcN(NS,3S,6S)] is also present.

Heparinase II-resistant tetrasaccharides (ΔU(4,5,0)-10 to ΔU(4,5,0)-13 and ΔU(4,6,0)-14 to ΔU(4,6,0)-16) were detected in all fractions. This is specific to BMH, since their content in BLH digests is limited, and they are absent from PMH. For the ΔU(4,5,0) tetrasaccharides, ΔIIs-IIIs_id_, ΔIIIs-IIIs_id_ and ΔIVs-Is_id_ can be anticipated, while ΔIs-IIIs_id_ and ΔIIIs-Is_id_ are likely for ΔU(4,6,0), knowing that ΔIIs-Is_id_ has been already confirmed by the action of Δ^4−5^ 2-*O*-sulfatase on ΔIs-Is_id_. It can be noted that in PMH as in BLH the main tetrasaccharides with reducing -Is_id_ are ΔIIs-Is_id_ and ΔIs-Is_id_, with no component that can be apparently attributed to ΔIIIs-Is_id_. The estimated tetrasaccharide content of heparinase II digests from BMH fractions are shown in Table 5.

**Table 5:** Quantification of ΔU(4,5,0) and ΔU(4,6,0) tetrasaccharide heparinase II-resistant building blocks (% w/w) for bovine mucosal heparin (BMH; starting heparin) affinity fractions using ion-pair liquid chromatography/mass spectrometry.

	**ΔU(4,5,0)**									
	**4**	**ΔIIs-IIs_glu_** ^**a**^	**ΔIIIs-IIs**_**glu**_^**b**^	**10**	**5^b^**	**11^b^**	**12^b^**	**ΔIs-IVs_**glu**_^**b**^**	**13^a^**	**6**
^*^				**ΔIVs-Is**_**id**_					**ΔIIs-IIIs**_**id**_	
**BMH**	**0.07**	**0.46**	**0.31**	**0.18**	**0.13**	**0.04**	**0.24**	**0.13**	**0.04**	**0.05**
**LA**	0.03	0.05	0.25	0.19	0.04	0.19	0.33	0.02		0.06
**HA1**	0.12	0.52	0.36	0.34	0.19	0.05	0.21	0.22	0.16	0.08
**HA2**	0.08	0.72	0.26	0.22	0.08	0.08	0.14	0.22	0.11	0.07
**HA3**	0.08	1.06	0.24	0.20	0.07	0.06	0.11	0.29	0.10	0.06
**HA4**	0.10	2.24	0.29	0.24	0.10	0.05	0.08	0.22	0.09	0.08
**HA5**	0.12	2.43	0.30	0.18	0.14	0.01	0.05	0.14	0.07	0.08
	Δ**U (4,6,0)**								Δ**U (4,7,0)**		**U (4,7,0)**
	**7** ^b^	Δ**Is-IIs_glu_^b^**	Δ**IIs-Is_id_**	**14** ^b^	**15** ^b^	**16** ^b^	**GlcNS,3S,6S-Is_id_**		Δ**Is-Is_id_^b^**	Δ**Is-Is_glu_^b^?**	**Is**_id_ **-Is_id_**
^*^				Δ**IIIs-Is_id_**		Δ**Is-IIIs_id_**				
**BMH**	**0.03**	**1.15**	**0.30**	**0.16**	**0.12**	**0.12**	**0.15**		**0.34**	**0.03**	**0.05**
**LA**	0.02	0.10	0.14	0.16	0.18	0.08	0.03		0.17	0.00	-
**HA1**	0.03	1.15	0.57	0.22	0.22	0.53	0.12		0.29	0.10	0.01
**HA2**	0.01	2.06	0.44	0.19	0.21	0.25	0.11		0.30	0.06	0.03
**HA3**	0.05	4.19	0.59	0.19	0.31	0.23	0.14		0.44	0.06	-
**HA4**	0.07	3.23	0.69	0.24	0.27	0.20	0.19		0.64	0.08	-
**HA5**	0.10	1.94	0.73	0.26	0.26	0.22	0.23		0.73	0.11	-

*Reference values: ΔIIs-IIs_glu_ and ΔIs-IIs_glu_ obtained from building block analysis on AS11 columns (part 2; to be published elsewhere)*.

a *Digested by Δ^4−5^ glycuronidase*.

b *Digested by Δ^4−5^ 2-O-sulfatase*.

* *Hypothesis of structure*.

ΔU(4,5,0)-11 was coeluted with ΔU(4,5,0)-5, but analysis of other BMH batches with a lower ΔU(4,5,0)-5 content shows that they are two different compounds. The ΔU(4,5,0)-11 in Table 5 was postulated on the assumption that ΔU(4,5,0)-5 appears to the same extent as in heparinases I+II+III digests (Table 4). ΔU(4,6,0)-14,15 and 16 are all sensitive to Δ^4−5^ 2-*O*-sulfatase, and 2-*O*-desulfated compounds are ΔU(4,5,0)-10, 13 and possibly 6. Within the six affinity fractions, the proportions of ΔU(4,6,0)-14,15 and 16 vary significantly, and remain unchanged after the action of Δ^4−5^ 2-*O*-sulfatase, but are detected as increasing amounts of the corresponding ΔU(4,5,0) tetrasaccharides. Thus, ΔU(4,5,0)-10, 13 and 6 are likely to be the 2-*O*-desulfated tetrasaccharides of ΔU(4,6,0)-14,16 and 15, respectively. Consequently, ΔU(4,5,0)-10, 13 and 6 are Δ^4−5^ 2-OH, and do not react with Δ^4−5^ 2-*O*-sulfatase; only ΔU(4,5,0)-13 is sensitive to Δ^4−5^ glycuronidase. We can thus assume that ΔU(4,5,0)-13 is 6-*O*-sulfated on its NR disaccharide, and corresponds to ΔIIs-IIIs_id_, and, consequently, ΔU(4,6,0)-16, ΔIs-IIIs_id_. ΔU(4,5,0)-10 is the only other heparinase II resistant tetrasaccharide which is Δ^4−5^ 2-OH, so that within the three available possibilities ΔIIs-IIIs_id_, ΔIIIs-IIIs_id_, ΔIVs-Is_id_, ΔU(4,5,0)-10 is necessarily ΔIVs-Is_id_ and ΔU(4,6,0)-14, its 2-*O*-sulfated derivative, ΔIIIs-Is_id_. For the last one, ΔU(4,6,0)-15, no other possibility remains if the configurations of the two disaccharides -IIIs_id_ and-Is_id_ are, as expected, iduronic. Similarly, both ΔU(4,5,0)-11 and 12 are Δ^4−5^ 2-*O*-sulfated, and only ΔIIIs-IIIs_id_ is available. To extend the structural limits, the configuration of the 3S sulfated disaccharide is the only parameter that can possibly be modified. The -IIs_id_ option can be rejected because tetrasaccharides 11-16 are detected in fractions where ΔIIs is absent, which means that they all have either Is or IIIs at their RE.

Consequently, the new structural options will necessarily have to be found in tetrasaccharides where the uronic acids of Is or IIIs are in the glucuronic configuration. Glucuronic 2-sulfate content is evidently low in bovine heparins, but not absent as in PMH (27, 48). The contents of higher tetrasaccharides such as ΔU(4,7,0), as shown in Table 5, support this contention. The main component, ΔIs-Is_id_, is the only one observed in LA fractions. However, in HA fractions (particularly HA1), a second is present with MS fragmentation identical to ΔIs-Is_id_. Both react with Δ^4−5^ 2-*O*-sulfatase, and if the number of sulfates is considered with the additional requirement of the 3-*O*-sulfate on the reducing disaccharide, the only possibility is the glucuronic configuration for Is in ΔIs-Is_glu_ [ΔHexUA(2S)-GlcN(NS,6S)-GlcA(2S)-GlcN(NS,3S,6S)]. Its proportion vs. ΔIs-Is_id_ is maximized for HA1 (1 to 3), and decreases in fractions of higher affinity for ATIII. While the same phenomenon is observed in BLH, only ΔIs-Is_id_ is detected in PMH. The 12 resistant ΔU(4,5,0) tetrasaccharides detected in the BMH HA5 fraction (Figure 8b) indicate further the remarkable structural diversity of BMH, where switching uronic configurations and consecutive 3-*O*-sulfated disaccharides may explain the various building blocks detected.

##### Hexasaccharide Building Blocks

**Figure 9:**
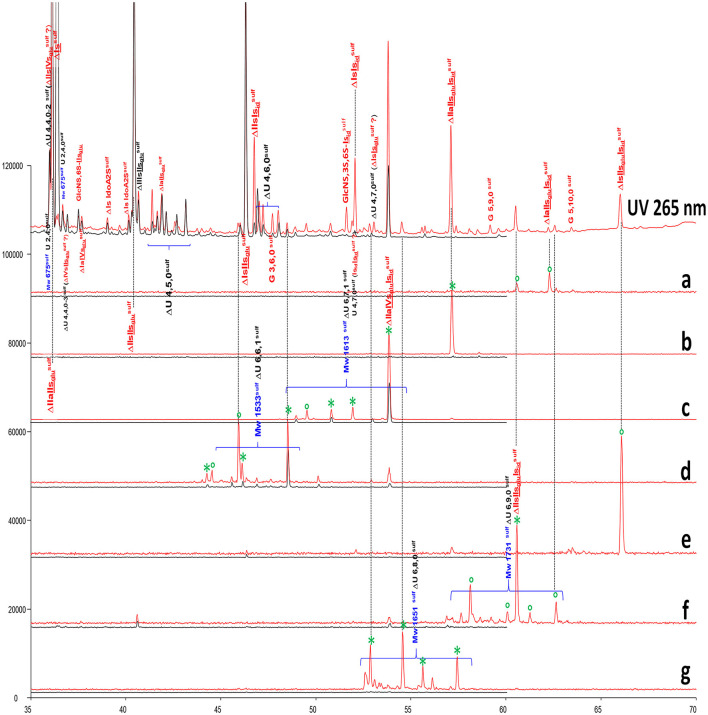
Comparison by ion-pair liquid chromatography/mass spectrometry (LC/MS) of heparinase I+II+III (**—**) and heparinase II (**—**) digests of bovine mucosal heparin high-affinity (HA5) fraction after sulfanilic reductive amination. **(a)** Reconstructed ion pair chromatograms (RIC) ΔU(6,9,1)^sulf^: m/z 1252.3; **(b)** RIC ΔU(6,8,1)^sulf^: m/z 1154.3; **(c)** RIC ΔU(6,7,1)^sulf^: m/z 1056.7; **(d)** RIC ΔU(6,6,1)^sulf^: m/z 959.2; **(e)** RIC ΔU(6,10,0)^sulf^: m/z 1271.6; **(f)** RIC ΔU(6,9,0)^sulf^: m/z 1173.3; **(g)** RIC ΔU(6,8,0)^sulf^: m/z 1075.7. *Digested by Δ^4−5^ glycuronidase; ° digested by Δ^4−5^ 2-*O*-sulfatase.

The chromatograms in Figure 3 show the presence of resistant hexasaccharides in heparinase II digests of BMH. Content increases with the ATIII affinity of the fraction (Supplementary Figure 9), so that, in HA4 and HA5 fractions, some were found to contain a full ATIII high-affinity pentasaccharide with an extra 3-*O*-sulfate on the last glucosamine of the pentasaccharide. Figure 9 shows digests of a BMH HA5 fraction with RIC's corresponding to the main hexasaccharides present in the fraction.

Structures were confirmed by LC/MS and by the action of Δ^4−5^ glycuronidase and Δ^4−5^ 2-*O*-sulfatase. Unknown hexasaccharides are probably heparinase II building blocks corresponding to the digestion-resistant precursors of tetrasaccharides such as ΔU(4,6,0)-8 and ΔU(4,6,0)-9, which contain an unsaturated 3S disaccharide.

The presence of these double 3-*O*-sulfated ATIII pentasaccharide sequences was already reported as highly probable (27) after the isolation of ΔIIa-IIs_glu_-Is_id_-Is_id_ from semuloparin (43). However, this is the first time that a straightforward analytical tool has been used to demonstrate this. In particular, the sulfate distribution of the ATIII binding sites with this type of sequence corresponds widely to conventional sequences with only one 3-*O*-sulfated glucosamine, and it is therefore logical to suggest that this extra 3-*O*-sulfate is obtained by secondary reaction with 3-*O*-sulfotransferases. This hypothesis was supported by the results of preliminary experiments in which 3-OST-1 (49) was applied to heparin.

## Conclusions

The present study re-examines the heparinase depolymerization of 3S (3-*O*-sulfated) heparin sequences, which determine the heparin anticoagulant activity. Additionally, in building block analyses, an important aspect of differentiation lies in the adequate recognition of 3S building blocks through their specificity to the heparin source. The well-characterized resistance to digestion of 3S disaccharides is due to the presence of the 3-*O*-sulfate moiety for heparinase II only; for heparinase I, resistance is not due to 3-*O*-sulfate, but depends rather on the structural environment, and particularly on the 2-OH-moiety in the preceding glucuronic acid when the sequence is the pentasaccharide ATIII binding site.

The detection in heparinases I+II+III digests of unsaturated 3S disaccharides is explained by the specific action of heparinase I on non-conventional 3S sequences. In heparinase II digests, unsaturated 3S disaccharides are not present and are eluted as tetra- or hexasaccharides where the 3-*O*-sulfate is located on the reducing glucosamine. Bovine intestinal heparin and its ATIII low- and high-affinity fractions were used to highlight the differences between heparinases I+II+III and heparinase II digests. Additional tagging by amino reduction of building blocks with sulfanilic acid was used to detect non-reducing ends and to improve chromatographic resolution.

It should be first specified that heparinase II selective digestion is not intended to replace heparinase I+II+III digestion, which remains the first-choice method for heparin analysis. However, these two analytical methods were used here primarily to show the structural diversity of the bovine intestine heparin largely consequent to its low 6-*O*-sulfation. In fact, six ΔU(4,4,0) and ΔU(4,5,0) 3S tetrasaccharides, only present in trace amounts in other heparin sources, were detected in heparinases I+II+III digests. Moreover, the two disaccharides ΔHexUA(2S)-GlcN(NS,3S) and ΔHexUA(2S)-GlcN(NS,3S,6S) detected in heparinases I+II+III digests generate at least 10 specific 3S tetrasaccharide building blocks in heparinase II digests. In fractions with highest affinity for ATIII, hexasaccharides containing the entire ATIII binding site with an extra 3-*O*-sulfate on the last glucosamine of the pentasaccharide have been identified. The double 3-*O*-sulfation of this type of sequence causes resistance to heparinase II digestion, and represents the first chromatographic analysis of an entire, biosynthetically modified ATIII binding site.

Finally, the purpose of this work was 2-fold. The first objective, described in this first part, was to explain the presence of 3S disaccharides in heparin digests through a cleavage on their non-reducing side by heparinase I thus correcting the misunderstood action of this enzyme for 3S sequences. The completion of the first part was to take advantage of this phenomenon and describe a new analytical procedure in heparin building block analyses, in which digests by heparinases I+II+III and heparinase II alone are compared, to improve the knowledge of the structural neighborhood of the 3S disaccharides.

The second objective, described in the second part (50), was to check the binding affinity to ATIII of the 3S disaccharides, which have still unknown biological functions. This was done across the comparison of six fractions of increasing affinity to ATIII for porcine intestine, bovine intestine and bovine lung heparins with building block analysis methods including sulfanilic tagging and heparinases I+II+III/heparinase II digestions. The distribution of 3S building blocks in the affinity fractions will be given for the three heparin sources with a special focus on 3S disaccharides, giving a first answer to their part in the binding to ATIII of heparin.

## Data Availability Statement

The original contributions presented in the study are included in the article/Supplementary Material, further inquiries can be directed to the corresponding author/s.

## Author Contributions

PM was responsible for all aspects of the study.

## Conflict of Interest

PM is an employee of Sanofi Chimie.

## Publisher's Note

All claims expressed in this article are solely those of the authors and do not necessarily represent those of their affiliated organizations, or those of the publisher, the editors and the reviewers. Any product that may be evaluated in this article, or claim that may be made by its manufacturer, is not guaranteed or endorsed by the publisher.

## Abbreviations

ATIII: Antithrombin III
NR: Non-reducing
BSA: Bovine Serum Albumin
NRE: Non-reducing end
BLH: Bovine lung heparin
NRS: Non-reducing side
BMH: Bovine mucosal heparin
PMH: Porcine mucosal heparin
GPC: Gel Permeation Chromatography
RE: Reducing end
HA: High Affinity
RIC: Reconstructed Ionic Current
HPTA: Heptylamine
RS: Reducing side
LA: Low Affinity
SAX: Strong Anion Exchange
LC/MS: Liquid Chromatography/Mass Spectrometry
TIC: Total Ionic Current
LMWH: Low-molecular-weight heparin
UPLC: Ultra Performance Liquid Chromatography
Mw: Mass average molecular weight
3S: 3-*O*-sulfated.
