# Supplementary material for: Heparinase Digestion of 3-O-Sulfated Sequences: Selective Heparinase II Digestion for Separation and Identification of Binding Sequences Present in ATIII Affinity Fractions of Bovine Intestinal Heparins
Source: Front Med (Lausanne). 2022 Mar 31;9:841726. doi: 10.3389/fmed.2022.841726 (PMC9009448; doi:10.3389/fmed.2022.841726)
Supplement: Supplementary file 1 [file Data_Sheet_1.pdf]

Supplementary Material

Heparinase digestion of 3-*O*-sulfated sequences: selective heparinase II digestion for separation and identification of binding sequences present in ATIII affinity fractions of bovine intestinal heparins

1 GPC follow-up of heparinase I digestion of heparin versus heparinase II

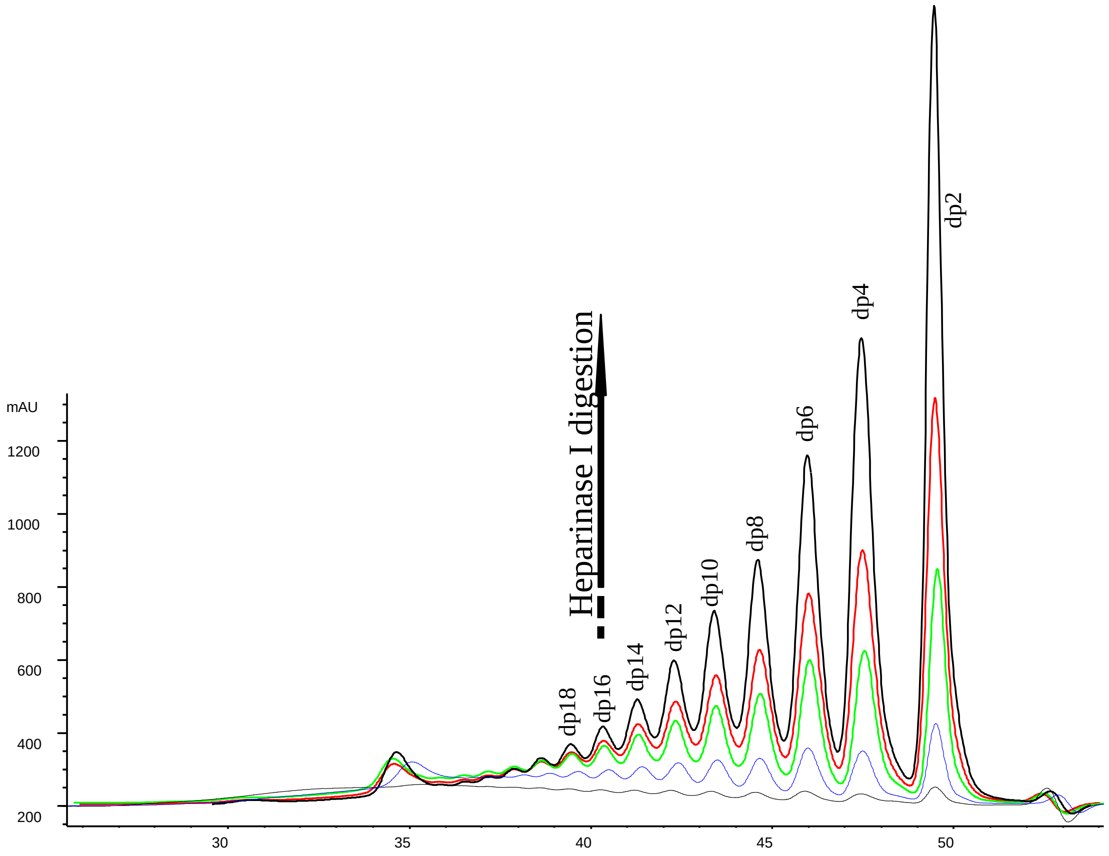

S-Figure 1: GPC follow up of heparin digestion by heparinase I (UV detection at 232nm); dp2: disaccharides, dp4: tetrasaccharides, dp6: hexasaccharides, dp8: octasaccharides, dp10: decasaccharides, dp12: dodecasaccharides, dp14: tetradecasaccharides, dp16: hexadecasaccharides, dp18: octadecasaccharides

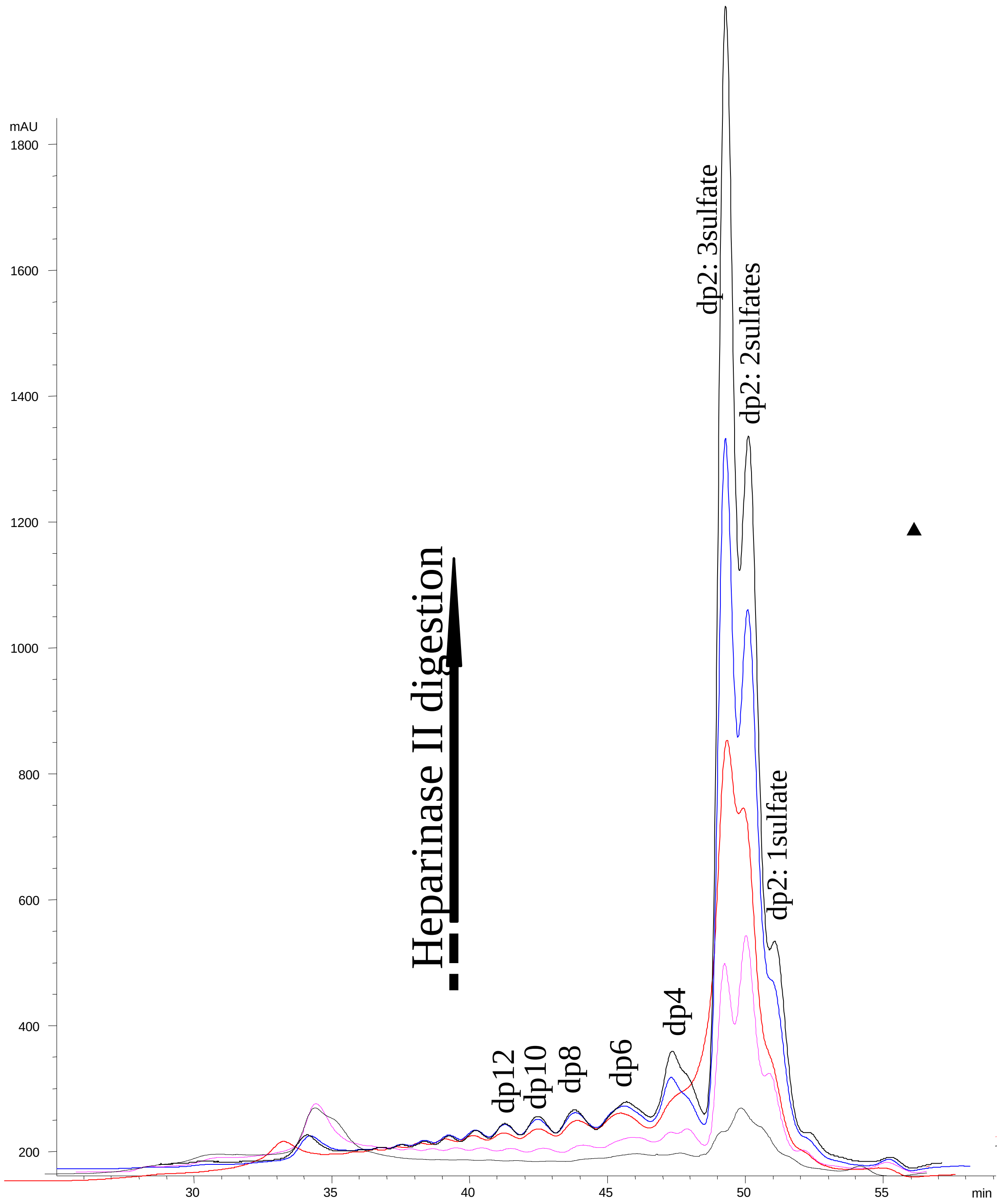

S-Figure 2: GPC follow up of heparin digestion by heparinase II (UV detection at 232nm); dp2: disaccharides, dp4: tetrasaccharides, dp6: hexasaccharides, dp8: octasaccharides, dp10: decasaccharides, dp12: dodecasaccharides, dp14: tetradecasaccharides, dp16: hexadecasaccharides, dp18: octadecasaccharides

2 NMR characterization of ΔIs-<u>Is</u>$_{id}$

1 g of an ATIII LA fraction of bovine lung heparin was digested with 3 I.U of heparinase I. The digest was injected on a GPC column (210 x 5cm) filled with BioGel P30 and circulated with $NaClO_4$ (0.2M). The trisaccharides to decasaccharides fractions were collected and desalted. The content of the tetrasaccharide fraction (265.2mg) was first fractionated on a CTA-SAX (1) semi-preparative column (250 x 21mm) filled with Hypersil BDS C_{18} 5µm and circulated at 20ml/min of H_2O with 0.002% methane sulfonic acid with a concentration gradient (0 to 2.5N) of sodium methane sulfonate 2.5M pH 3. The column temperature was set at 55°C. 30 to 45mg of the tetrasaccharide fraction were injected at each run (figure 3S)

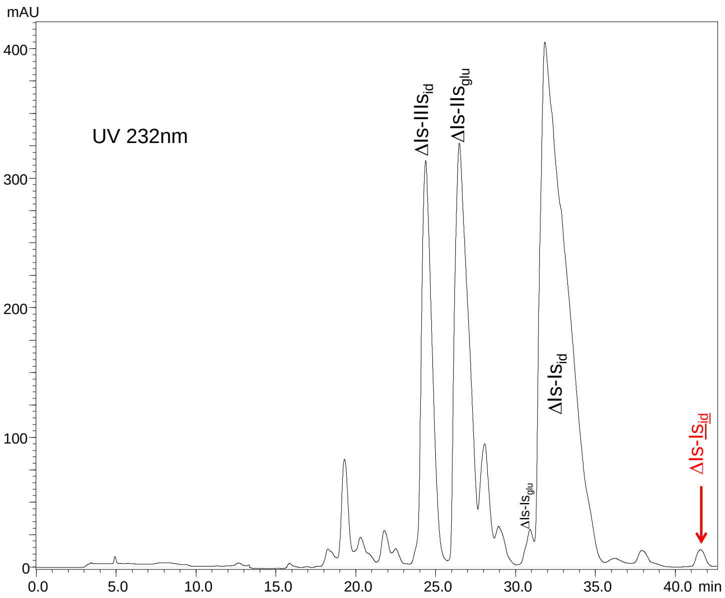

S-Figure 3: Semi-preparative separation on a CTA-SAX column fraction of a tetrasaccharide fraction of ATIII LA of bovine Lung heparin digested by heparinase I

The fraction corresponding to ΔIs-<u>Is</u>$_{id}$ was in a second step, reinjected on an AS11(Dionex, Ile-de-France, France) SAX columns (250 x 21mm).

---

[1] Mourier PAJ, Viskov C. Chromatographic analysis and sequencing approach of heparin oligosaccharides using cetyltrimethylammonium dynamically coated stationary phases. Anal Biochem (2004) 332(2):299-313. doi: 10.1016/j.ab.2004.06.020.

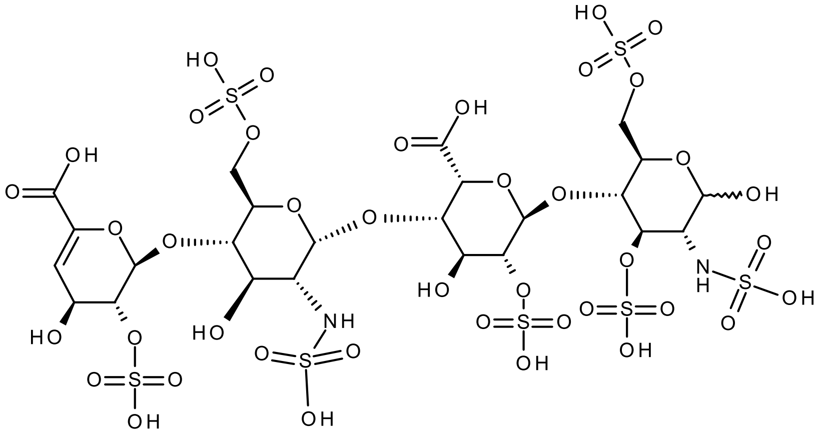

d c b a

ΔIs $\underline{Is_{id}}$

NMR experiments were run at 600MHz using Bruker Avance instrument (Bruker Biospin) equipped with a 5mm inverse cryoprobe. About 0.2 milligram of the purified oligosaccharide was dissolved in a 3 mm tube containing 150µL of 99.9% D2O. The temperature was set at 25°C and pre-saturation of the residual water signals was used to improve signal to noise ratio. Spectral parameters for 1H spectra include 128 transients, approximately 30◦ pulse width, acquisition time of at least one second, time between transients of three seconds and a spectral window of 9600 Hz. Reference was set at 0.00ppm for the TSP-d4 signal. Proton and Carbon assignments were obtained using 2D TOCSY and 1H-13C HSQC spectra.

Proton and Carbon Chemical shifts in ppm (600MHz, D_2O, 25°C):

S-Table 1: Proton and carbon chemical shifts for ΔIs-$\underline{Is_{id}}$

Residue	1	2	3	4	5	6,6’
a	5.42 (94.1)	3.47 (59.7)	4.49 (78.3)	4.06 (74.9)	4.19 (72.4)	4.28/4.50
b	5.17	4.32 (80.4)	4.20 (79.4)	4.11 (73.8)	4.86 (73.6)	-
c	5.54 (98.6)	3.30 (60.4)	3.63 (72.3)	3.84 (81.1)	4.11 (71.7)	4.27/4.37
d	5.51	4.64 (77.4)	4.32 (65.7)	5.99	-	-

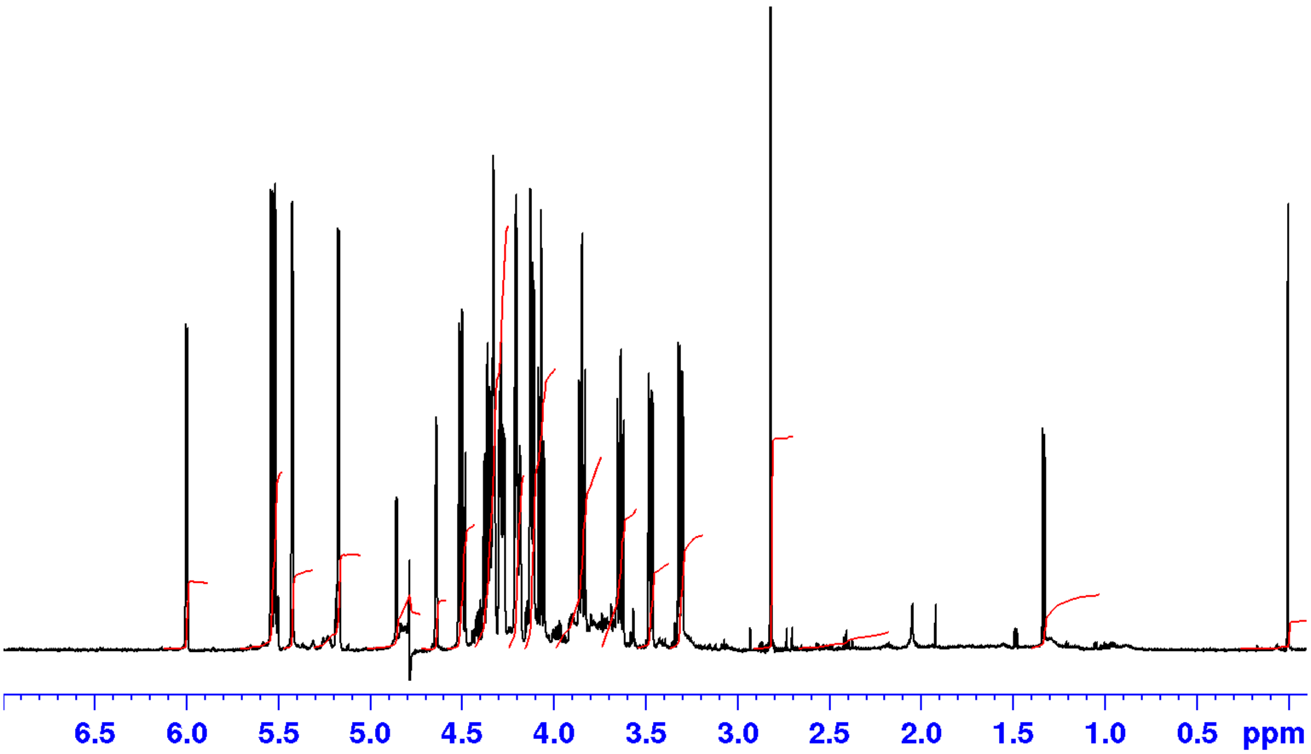

S-Figure 4. 1H spectrum of ΔIs-Is$_{id}$ (D_2O, 25°C, 500 MHz)

3 NMR characterization of ΔIs-IIa$_{id}$-IIs$_{glu}$-IIs$_{glu}$-IIs$_{glu}$

ΔIs-IIa$_{id}$-IIs$_{glu}$-IIs$_{glu}$-IIs$_{glu}$ was isolated from an ATIII HA5 high affinity fraction (1g) from a porcine mucosa heparin. Briefly, the fraction was partially depolymerized by heparinase I. The depolymerized fraction was then fractionated by ATIII affinity chromatography. The high affinity fraction (338mg) was collected and injected on a GPC column (210 x 5cm) filled with BioGel P30 and circulated with $NaClO_4$ (0.2M). Hexasaccharide to dodecasaccharide fractions were collected. The decasaccharide fraction (11.7mg) was purified on a CTA-SAX column (150 x 10mm) Ascentis C_{18} 2.7µm Supelco® and then on a AS11 (Dionex, Ile-de-France, France) SAX columns (250 x 21mm).

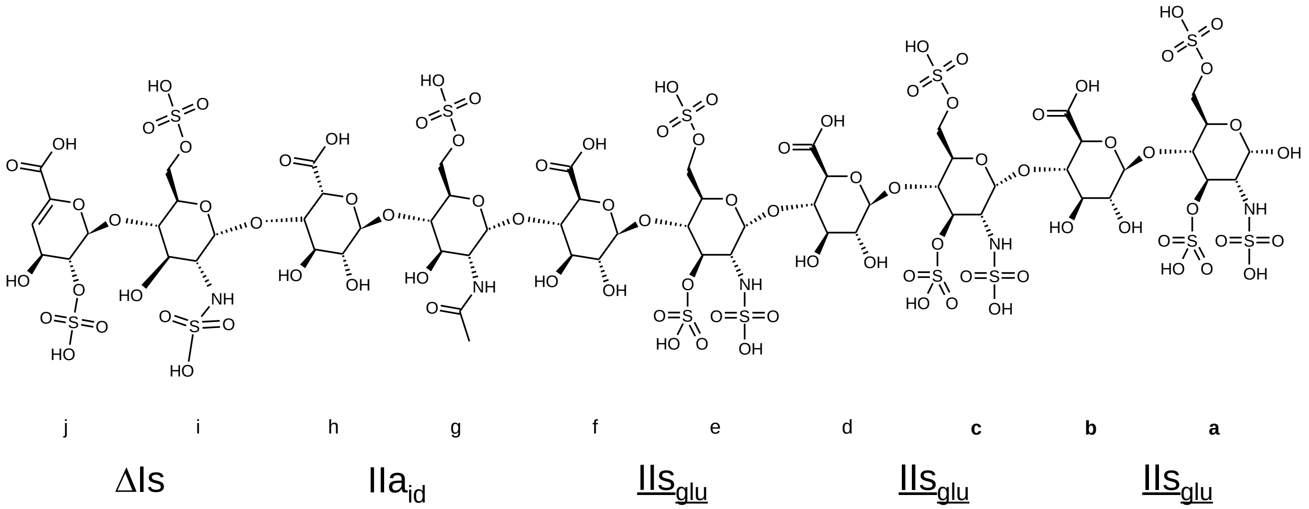

NMR experiments were run at 500 MHz using Bruker Avance instrument (Bruker Biospin) equipped with a 5 mm inverse cryoprobe. About 0.1 mg of the purified oligosaccharide was dissolved in a 3 mm tube containing 150µL of 99.9% D_2O. The temperature was set at 30°C and pre-saturation of the residual water signals was used to improve signal-to-noise ratio. Spectral parameters for 1H spectra include 128 transients, approximately 30 pulse width, acquisition time of at least one second, time between transients of three seconds and a spectral window of 9600 Hz. Reference was set at 0.00 parts per million (ppm) for the TSP-d4 signal. Proton assignment was obtained using 2D TOCSY and COSY spectra.

Proton Chemical shift in ppm (500 MHz, D_2O, 25°C):

S-Table 2: Proton and carbon chemical shifts for ΔIs-IIa_{id}-$\underline{IIs_{glu}}$-$\underline{IIs_{glu}}$-$\underline{IIs_{glu}}$

Residue	1	2	3	4	5	6,6	Acetyl
a	5.45	3.46	4.52	4.02	4.24	4.22/4.35	
b	4.65	3.43	3.82	3.87	3.86	-	
c	5.59	3.46	4.41	3.98	4.07	4.22/4.50	
d	4.63	3.43	3.82	3.88	3.86	-	
e	5.58	3.45	4.40	3.97	4.08	4.22/4.50	
f	4.61	3.38	3.69	3.76	3.76	-	
g	5.39	3.93	3.79	3.73	4.02	4.21/4.34	2.05
h	5.00	3.78	4.07	4.12	4.82	-	
i	5.35	3.28	3.64	3.84	3.98	4.22/4.37	
j	5.50	4.63	4.33	5.99	-	-	

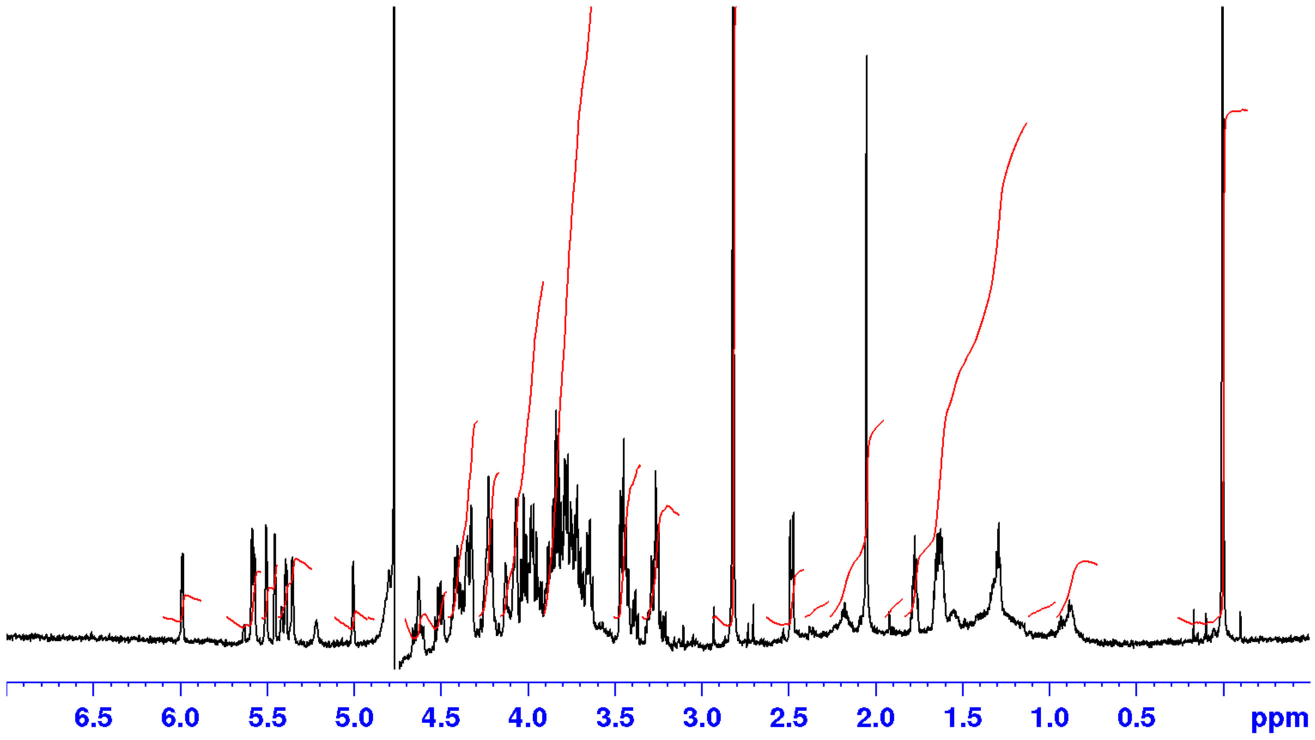

S-Figure 5. 1H spectrum of ΔIs-IIa$_{id}$-<u>IIs</u>$_{glu}$-<u>IIs</u>$_{glu}$-<u>IIs</u>$_{glu}$ (D_2O, 25°C, 500 MHz)

4 Sequencing of ΔIs-IIa$_{id}$-<u>IIs</u>$_{glu}$-<u>IIs</u>$_{glu}$-<u>IIs</u>$_{glu}$

The sequencing experiment is shown in S-Figure 6. The oligosaccharide sequenced corresponds to ΔIs-IIa$_{id}$-<u>IIs</u>$_{glu}$-<u>IIs</u>$_{glu}$-<u>IIs</u>$_{glu}$ previously identified by NMR. Chromatograms of digests were produced using an AS11 (Dionex) SAX columns.

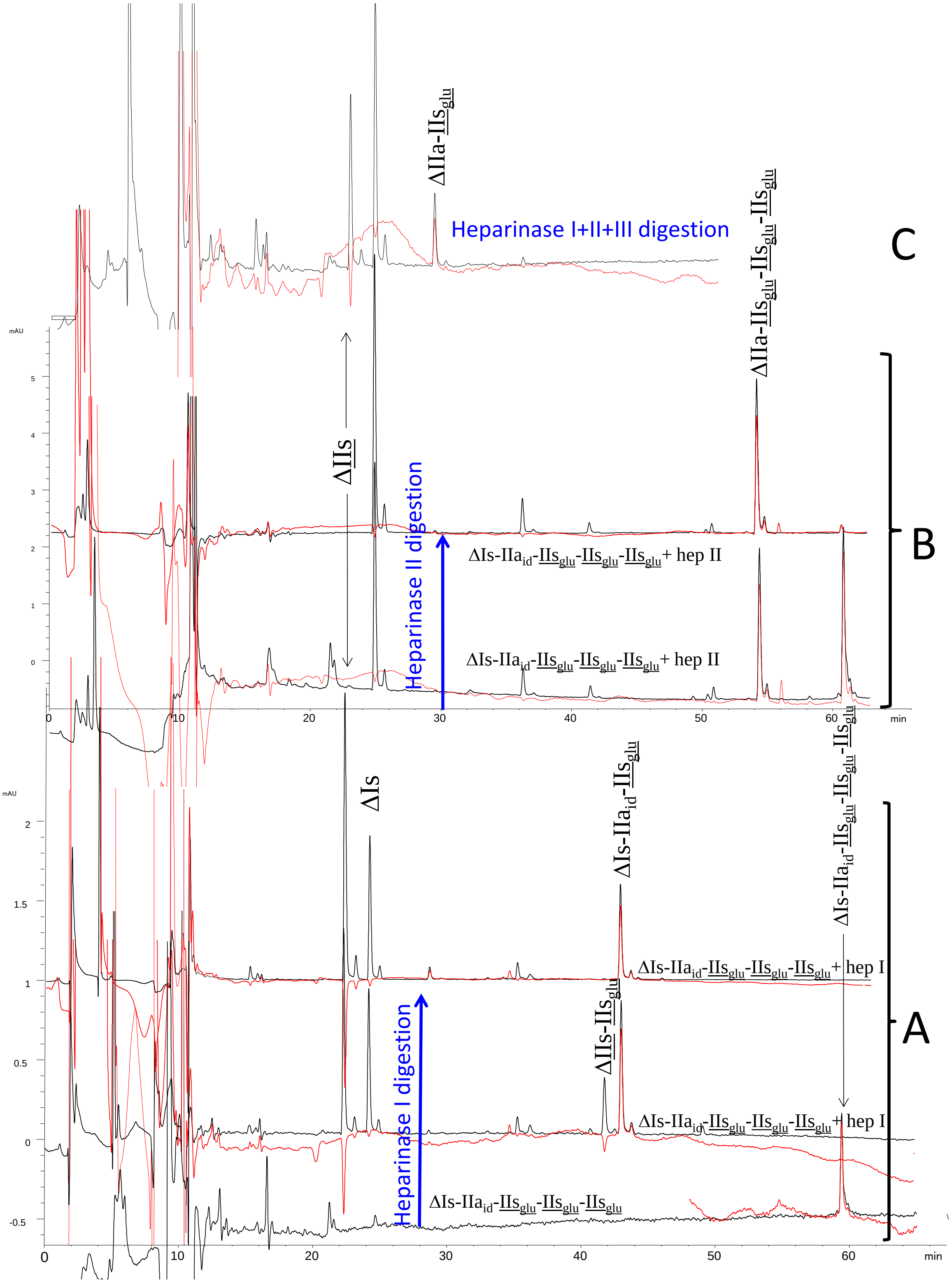

S-Figure 6. Sequencing on AS11 chromatograms of the decasaccharide ΔIs-IIa$_{id}$-IIs$_{glu}$-IIs$_{glu}$-IIs$_{glu}$ (black line: UV 232 nm; red line: UV 202 nm-242 nm) by digestion with heparinases. A) Follow up of depolymerization by heparinase I, B) Follow up of depolymerization by heparinase II, C) Depolymerization by the heparinase mixture

Interestingly, the action of heparinase II on this decasaccharide gives a mixture of ΔIs with the octasaccharide ΔIIa-IIs$_{glu}$-IIs$_{glu}$-IIs$_{glu}$. In this state, the reaction is at completion, confirming the inability of heparinase II to cleave ↓IIs$_{glu}$ bonds. The action of heparinase I reveals two cleavable sites: ΔIs-IIa$_{id}$-Is$_{glu}$↓ IIs$_{glu}$↓ IIs$_{glu}$, so that a mixture of ΔIs-IIa$_{id}$-IIs$_{glu}$, ΔIIs-IIs$_{glu}$ and ΔIIs is obtained. ΔIIs-IIs$_{glu}$ is then entirely depolymerized into ΔIIs.

LC/MS chromatograms of the starting decasaccharides ΔIs-IIa$_{id}$-IIs$_{glu}$-IIs$_{glu}$-IIs$_{glu}$ and the digests by heparinase I and II are shown on S-Figure 7. Experimental conditions are identical to those previously described (2).

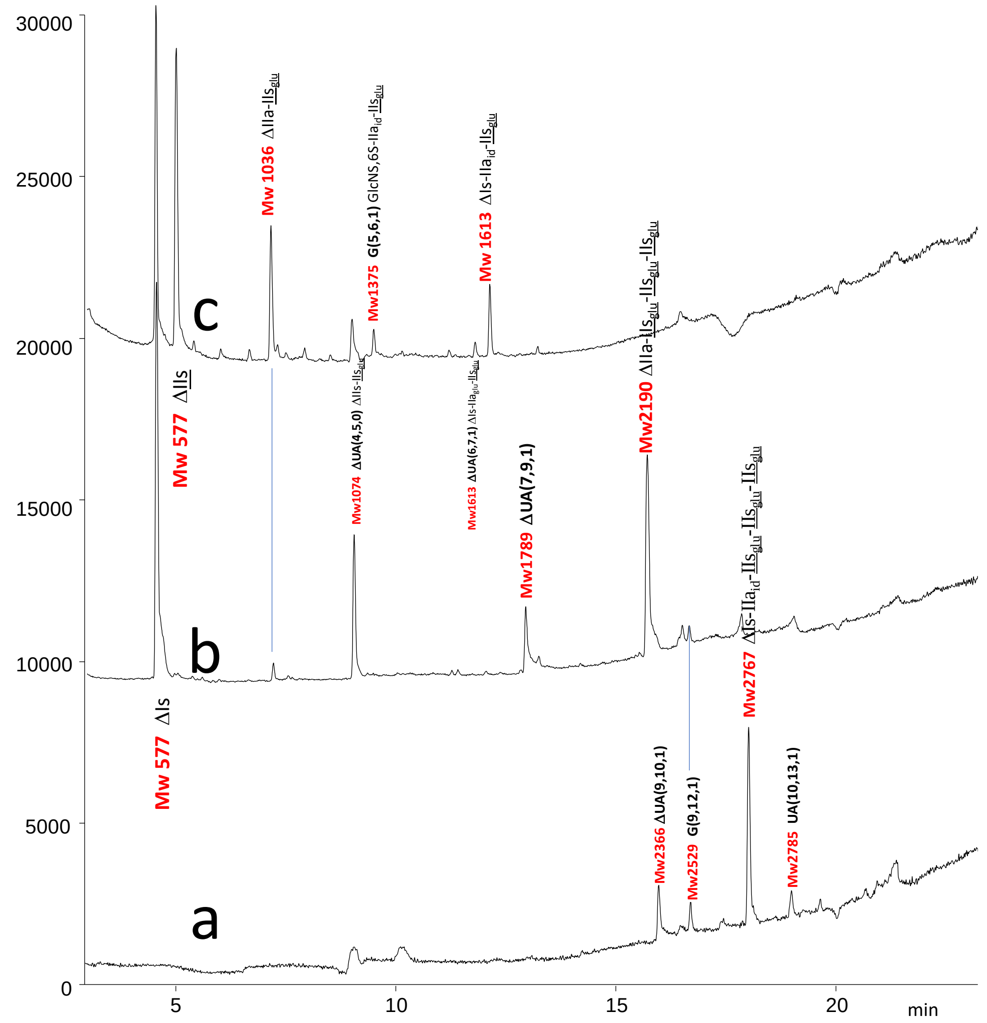

S-Figure 7. Sequencing of the decasaccharide ΔIs-IIa$_{id}$-IIs$_{glu}$-IIs$_{glu}$-IIs$_{glu}$: LC/MS follow up: a): TIC of the starting decasaccharides; b) TIC of the depolymerization by heparinase II; c) TIC of the depolymerization by heparinase I

---

[2] Mourier PAJ, Guichard OY, Herman F, Sizun P, Viskov C. New Insights in Thrombin Inhibition Structure-Activity Relationships by Characterization of Octadecasaccharides from Low Molecular Weight Heparin. Molecules (2017) 22(3):428. doi: 10.3390/molecules22030428.

5 m/z correspondence for generation of reconstructed ion chromatograms (figure 3)

S-Table 3: Correspondence of m/z values in Figure 3

Unsaturated disaccharides: m/z 535.14+573.07+615.094_615.2+652.906_653.05+695+848.1+1043.2				
m/z	Ion	Structure	Mw (Da)	Mw-Sulfanilic (Da)
535.14	(Mw-H)$^-$	ΔIVa	536	379
573.07	(Mw-H)$^-$	ΔIVs	574	417
615.15	(Mw-H)$^-$	ΔIIa, ΔIIIa,	616	459
653	(Mw-H)$^-$	ΔIIs, ΔIIIs,	654	497
695	(Mw-H)$^-$	ΔIa	696	539
848.1	(Mw+HPTA-H)$^-$	ΔIs	734	577
1043.2	(Mw+2HPTA-H)$^-$	ΔIs	814	657
Non-reducing end disaccharides: m/z 591.1+671.1+866.2+1061.3				
m/z	Ion	Structure	Mw (Da)	Mw-Sulfanilic (Da)
591.1	(Mw-H)$^-$	U(2,1,0)	592	435
671.1	(Mw-H)$^-$	U(2,2,0)	672	515
866.2	(Mw+HPTA-H)$^-$	Is$_{id}$, U(2,3,0)	752	595
1061.3	(Mw+2HPTA-H)$^-$	U(2,4,0)	832	675
Acetylated tetrasaccharides: m/z 555.6+595.6+635.5+515.6+952.2				
m/z	Ion	Structure	Mw (Da)	Mw-Sulfanilic (Da)
952.2	(Mw-H)$^-$	ΔIVa-IVs, ΔU(4,1,1)	953	796
515.5	(Mw-2H)$^{2-}$	ΔU(4,2,1)	1033	876
555.6	(Mw-2H)$^{2-}$	ΔIIa-IVs$_{glu}$, ΔU(4,3,1)	1113	956
595.6	(Mw-2H)$^{2-}$	ΔIIa-IIs$_{glu}$, ΔU(4,4,1)	1193	1036
635.5	(Mw+HPTA-H)$^-$	ΔIa-IIs$_{glu}$	1273	1116
Sulfated tetrasaccharides: m/z 614.5+712 +574.6+534.6+672.6+809.6				
m/z	Ion	Structure	Mw (Da)	Mw-Sulfanilic (Da)
574.6	(Mw-2H)$^{2-}$	ΔU(4,4,0)	1151	994
614.2	(Mw-2H)$^{2-}$	ΔIIs-IIs$_{glu}$, ΔU(4,5,0)	1231	1074
672.6	(Mw+HPTA-2H)$^{2-}$ +1	ΔIIs-IIs$_{glu}$, ΔU(4,5,0)	1232	1075
712	(Mw+HPTA-2H)$^{2-}$	ΔIs-IIs$_{glu}$, ΔU(4,6,0)	1311	1154
809.6	(Mw+2HPTA-2H)$^{2-}$	ΔIs-Is$_{id}$, ΔU(4,7,0)	1391	1234

Acetylated hexasaccharides: m/z 724.156_724.3+764.1+861.7+959.2+1056.8 +1154.3;

m/z	Ion	Structure	Mw (Da)	Mw-Sulfanilic (Da)
724.2	$(Mw\text{-}2H)^{2-}$	ΔU(6,3,1)	1450	1293
764.1	$(Mw\text{-}2H)^{2-}$	ΔU(6,4,1)	1530	1373
861.7	$(Mw\text{+}HPTA\text{-}2H)^{2-}$	ΔU(6,5,1)	1610	1453
959.2	$(Mw\text{+}2HPTA\text{-}2H)^{2-}$	ΔU(6,6,1)	1690	1533
1056.8	$(Mw\text{+}3HPTA\text{-}2H)^{2-}$	ΔIIa-$\underline{IVs_{glu}}$-$\underline{Isi_{d}}$, ΔU(6,7,1)	1770	1613
1154.3	$(Mw\text{+}4HPTA\text{-}2H)^{2-}$	ΔIIa-$\underline{IIs_{glu}}$-$\underline{Isi_{d}}$, ΔU(6,8,1)	1850	1693

Sulfated hexasaccharides: m/z 880.6+978.2+1133.3.

m/z	Ion	Structure	Mw (Da)	Mw-Sulfanilic (Da)
880.6	$(Mw\text{+}HPTA\text{-}2H)^{2-}$	ΔU(6,6,0)	1648	1491
978.2	$(Mw\text{+}2HPTA\text{-}2H)^{2-}$	ΔU(6,8,0)	1728	1571
1133.3	$(Mw\text{+}4HPTA\text{-}2H)^{2-}$	ΔU(6,7,0)	1808	1651

6 LC/MS chromatograms of heparinase I+II+III and heparinase II digests of heparin affinity fractions

See S-Figure 8.

7 LC/MS chromatograms of heparinase II digests of heparin affinity fractions

See S-Figure 9.

frontiers

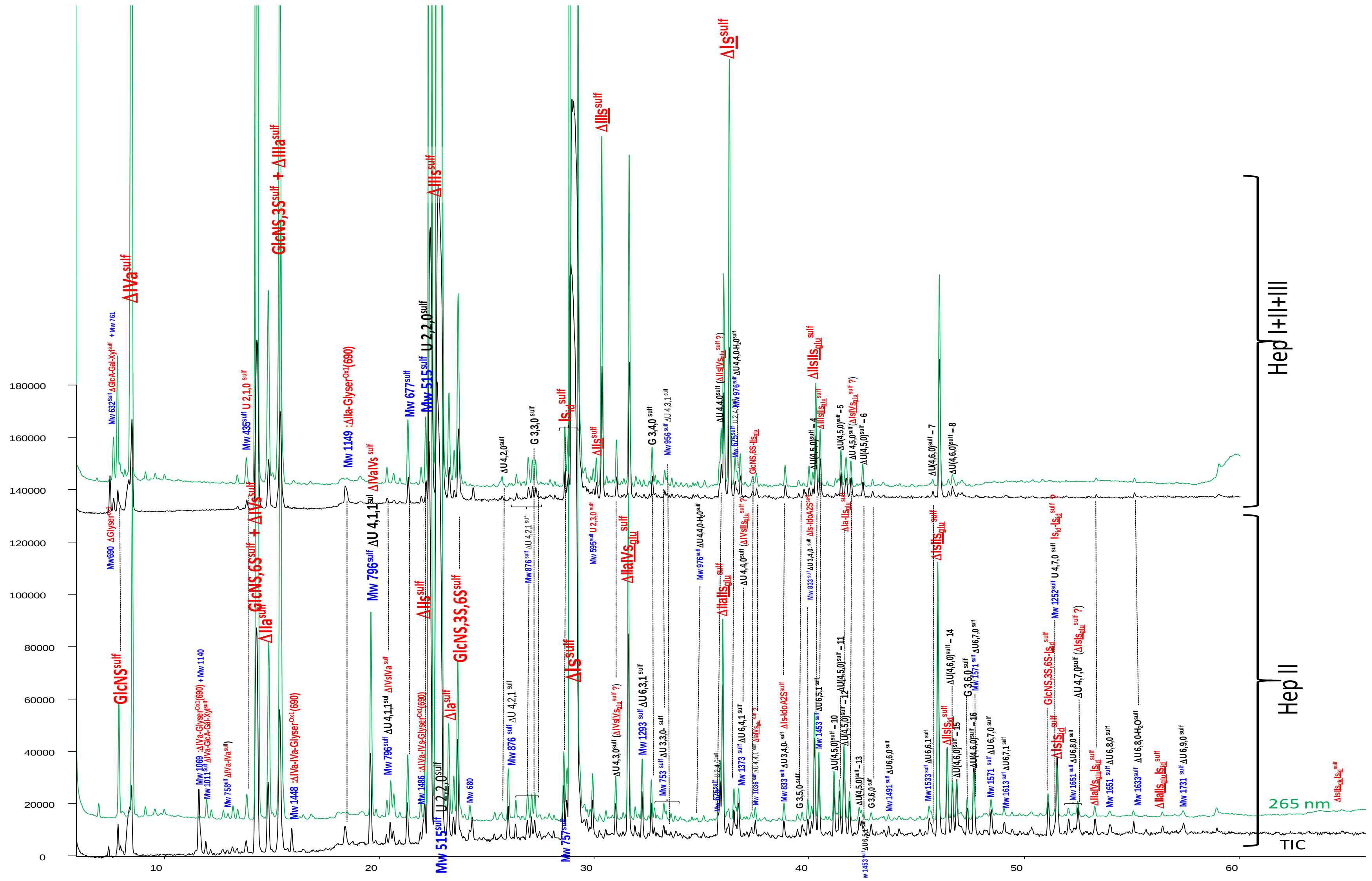

S-Figure 8: Comparison using LC/MS ion pair chromatography of BMH digests by the heparinase mixture versus heparinase II (Detection: — 265 nm; — TIC).

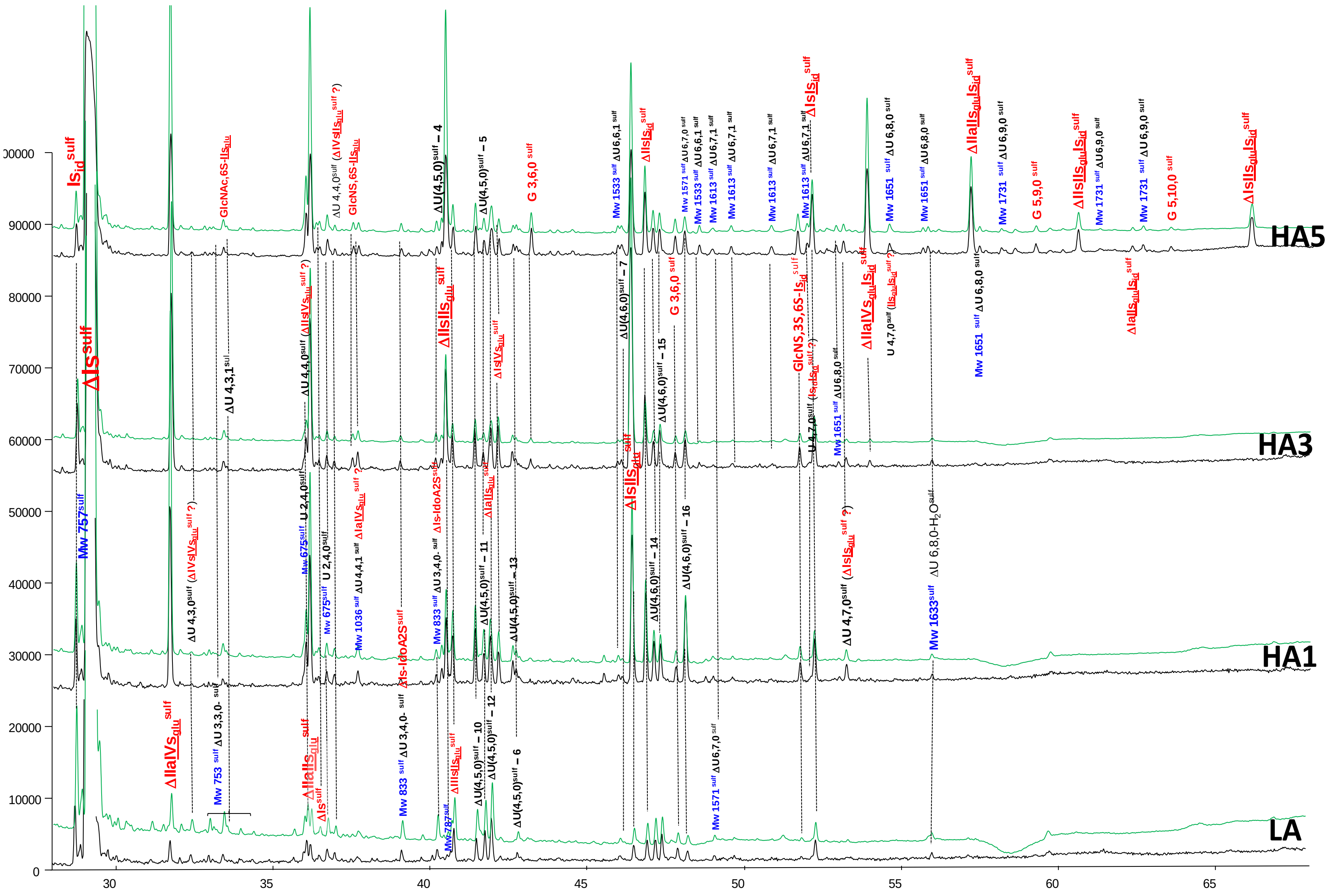

S-Figure 9: LC/MS chromatogram using ion-pair method of heparinase II digests from BMH affinity fractions with sulfanilic tagging. Detection: — 265 nm; — TIC
